# Cardiomyocyte depolarization triggers NOS-dependent NO transient after calcium release, reducing the subsequent calcium transient

**DOI:** 10.1007/s00395-021-00860-0

**Published:** 2021-03-17

**Authors:** Matias Mosqueira, Roland Konietzny, Carolin Andresen, Chao Wang, Rainer H.A. Fink

**Affiliations:** 1 Cardio-Ventilatory Muscle Physiology Laboratory, Institute of Physiology and Pathophysiology, University Hospital Heidelberg, Im Neuenheimer Feld 326, R. 305, 69120 Heidelberg, Germany; 2 Medical Biophysics Unit, Institute of Physiology and Pathophysiology, University Hospital Heidelberg, 69120 Heidelberg, Germany; 3 Heidelberg Institute for Stem Cell Technology and Experimental Medicine (HI-STEM gGmbH), Heidelberg, Germany; 4 Division of Stem Cells and Cancer, German Cancer Research Center (DKFZ), Heidelberg, Germany; 5 Cardiovascular Department, Wuhan No. 1 Hospital, Hubei, China

**Keywords:** Nitric oxide, Calcium transient, Cardiomyocyte, Duchenne muscular dystrophy, MDX, NO-ON, Rhod-2, Fluo-4

## Abstract

Cardiac excitation–contraction coupling and metabolic and signaling activities are centrally modulated by nitric oxide (NO), which is produced by one of three NO synthases (NOSs). Despite the significant role of NO in cardiac Ca^2+^ homeostasis regulation under different pathophysiological conditions, such as Duchenne muscular dystrophy (DMD), no precise method describes the production, source or effect of NO through two NO signaling pathways: soluble guanylate cyclase-protein kinase G (NO-sGC-PKG) and S-nitrosylation (SNO). Using a novel strategy involving isolated murine cardiomyocytes loaded with a copper-based dye highly specific for NO, we observed a single transient NO production signal after each electrical stimulation event. The NO transient signal started 67.5 ms after the beginning of Rhod-2 Ca^2+^ transient signal and lasted for approximately 430 ms. Specific NOS isoform blockers or NO scavengers significantly inhibited the NO transient, suggesting that wild-type (WT) cardiomyocytes produce nNOS-dependent NO transients. Conversely, NO transient in mdx cardiomyocyte, a mouse model of DMD, was dependent on inducible NOS (iNOS) and endothelial (eNOS). In a consecutive stimulation protocol, the nNOS-dependent NO transient in WT cardiomyocytes significantly reduced the next Ca^2+^ transient via NO-sGC-PKG. In mdx cardiomyocytes, this inhibitory effect was iNOS- and eNOS-dependent and occurred through the SNO pathway. Basal NO production was nNOS- and iNOS-dependent in WT cardiomyocytes and eNOS- and iNOS-dependent in mdx cardiomyocytes. These results showed cardiomyocyte produces NO isoform-dependent transients upon membrane depolarization at the millisecond time scale activating a specific signaling pathway to negatively modulate the subsequent Ca^2+^ transient.

**Supplementary Information:**

The online version contains supplementary material available at 10.1007/s00395-021-00860-0.

## Introduction

Cardiac excitation–contraction coupling (ECC) is the central physiological process of Ca^2+^-mediated contractility in the heart [9]. The transient Ca^2+^ released during the ECC is also responsible for the activation of kinases, phosphatases, calmodulin and other signaling pathways [9], such as NO, an active biomolecular gas with a 1-s half-life [50, 87, 89, 109, 110]. The action of NO is mediated by soluble guanylate cyclase (sGC), which activates protein kinase G (PKG), resulting in the phosphorylation of a series of proteins, such as the l-type Ca^2+^ channel and ryanodine receptor [50, 69, 87, 89]. Alternatively, NO also modifies protein activity by reacting with specific sulfur moieties from cysteine residues, forming *S*-nitrosylations (SNOs) [69, 93, 110]. The cardiac production of NO is mediated by one of three NO synthase isoforms expressed under normal conditions: neuronal (nNOS), inducible (iNOS) or endothelial (eNOS) [10, 30, 57, 69, 88, 105, 109, 110]. The three isoforms are expressed differentially and heterogeneously, varying in intensity and location between ventricles and in each muscle layer of the ventricle [12, 13]. One key differential characteristic of these three isoforms is related to their activation. Furthermore, nNOS and eNOS are transiently activated once Ca^2+^ released during the ECC binds to calmodulin (Ca^2+^-dependent NOS activation) [69, 101], while iNOS activation is activated through a FAK-PI(3)K-AKT signaling pathway dependent on contractility and independent of Ca^2+^ [16]. Indeed, iNOS activity summed to approximately one-third of total NOS activity in sham pig hearts and even higher after ischemia [34]. Under pathological conditions, the expression levels and activity of three NOS isoforms change accordingly, thus altering NO production [34, 55, 112]. The relevance of NO modulatory function in the ECC has been extensively exemplified in Duchenne muscular dystrophy (DMD), a lethal genetic disease characterized by the absence of dystrophin [26, 36], a large protein stabilizing the tissue structure by connecting F-actin and the extracellular matrix [69]. Dystrophin is associated with various essential regulatory processes of the ECC, such as Ca^2+^ homeostasis and NO production [37, 98, 100]. Histological observations of muscle degeneration, muscular fibrosis, respiratory insufficiency and cardiomyopathy in affected boys have been confirmed by electrocardiogram, echocardiograms, and heart variability analyses [53, 60, 66, 75]. The cellular mechanism of cardiomyopathy in DMD patients involves imbalanced Ca^2+^ and NO signaling, which leads to the replacement of healthy tissue in very discrete and localized small areas referred to as microinfarctions [1, 83]. As described in DMD patients, 10-month-old mdx mice exhibit advanced signs of cardiomyopathy, including fibrosis, inflammation, and macrophage infiltration, resulting in reduced contractility, reduced ejection fraction, abnormal left ventricle relaxation and reduced ventricular compliance [45, 79]. Cardiomyocytes from mdx mice exhibit reduced expression and activity levels of the neuronal isoform of NO synthase (nNOS) and thus reduced production of NO [69, 82, 97].

Multiple signs of cardiomyopathy in DMD patients and mdx mice are reverted with the administration of the NO precursor l-arginine, NO donors, nitrate supplementation, phosphodiesterase-5 blockers, and or overexpression of nNOS in an mdx mouse model [97]. Another relevant aspect in the DMD is the formation of reactive oxygen species (ROS) during the developing of the disease inducing NO production and transforming NO into peroxynitrite (ONOO^−^), recognized as a major contributor to myocardial depression [81]. Reactive oxygen species (ROS), such as H_2_O_2_ and superoxide, induces indirectly the expression of eNOS and nNOS, and therefore, the NOS-derived NO [4, 114]. Furthermore, nNOS is induced by the interplay between angiotensin II type 1 receptor and Angiotensin II type 2 receptor, mediated by NADPH oxidase and ROS-dependent eNOS activity in cardiac myocytes (from Ref. [114]). In addition, nNOS-derived NO also modulates intracellular redox status and ROS-dependent downstream effects in the myocardium by targeting multiple cardiac oxidases [114]. Therefore, the described above evidence suggests a mechanism to explain ROS-dependent NO production.

NO has been measured using indirect methods on an extended time scale of the cellular production of l-citrulline, cGMP, peroxynitrites, nitrates and nitrites [29, 74, 109]. However, due to its short half-life, the low specificity of NO fluorescence probes in comparison to its byproducts and the imprecision of the intracellular NO production time scale result in large margins of error, thus influencing results and interpretations [35, 71, 72]. The chronoamperometric system using a Nafion/ porphyrin-coated carbon electrode is the most accurate method to measure extracellular NO oxidation [35, 42, 68] and is capable of recording transient NO production during systole in both the rat endocardium and the rabbit endocardium and myocardium [76], but it still imprecisely determines the amount of NO produced intracellularly [19, 44, 106]. Consequently, it has not been possible to determine the real time scale of endogenous NO production, its effect and the signaling pathway activated during cardiomyocyte ECC. Therefore, we hypothesized that NOS-dependent NO would be transiently produced during the ECC accordingly to the pathophysiological alteration, modulating the Ca^2+^ transient through different signaling pathways. As a first approach, we addressed the open question of the role of NO during ECC by measuring NO production using a new copper-based dye specific to NO [62–64] in cardiomyocytes isolated from 12-month-old wild-type (WT) and mdx mice. Upon electrical stimulation, we detected a single NO transient in both WT and mdx cardiomyocytes 67.5 ms after the beginning of the Ca^2+^ transient, and it persisted for 430 ms. The NO transient was reduced using specific NOS isoform blockers or NO scavengers. The NO transient produced in WT cardiomyocytes reduced the next Ca^2+^ transient via the nNOS-NO-sGC-PKG pathway, while in mdx cardiomyocytes, the inhibitory effect was eNOS- and iNOS-dependent via the SNO pathway. These results showed isoform-dependent NO transient production after each cardiomyocyte membrane depolarization, exerting its modulatory effect on the next Ca^2+^ transient according to the pathophysiological condition.

## Materials and methods

### Mice

Wild type (C57BL/10ScSn) and MDX (C57BL/10ScSn-Dmd^mdx^/J) age-matched (average age 54 weeks) male mice were used for the experiments. All experiments were approved by the ethics committee of the University of Heidelberg Interfaculty Biomedical Research Facility (T-83/14 and T-21/17) and according to the guidelines of the Regierungspräsidium Karlsruhe of State of Baden- Wuerttemberg.

### Cell isolation

Single cardiomyocyte isolation using Langendorff perfusion set-up was previously described [56, 67, 111] with few modifications (Supplemental Information Material & Method). The isolated heart was cannulated in between the atria for the Perfusion Solution (in mM: 135 NaCl, 4 KCl, 1 MgCl_2_, 10 HEPES, 0.33 NaH_2_PO_4_, 10 Glucose, 20 2,3-butanedione monoxime BDM, 5 Taurine, pH 7.2) connected to the 37 °C pre-warmed Langendorff set-up. After 5 min of Perfusion Solution, the heart was perfused with Digestion Solution (Collagenase D, Roche, cat no. 11088858001, 0.36 mg/g of mouse; Collagenase B, Roche, cat no. 11088807001, 0.48 mg/g and Protease from *Streptomyces griseus* type XIV, Sigma-Aldrich, cat no. P5147-100MG, 0.06 mg/g) for 20–30 min. After digestion, the heart was mechanically dissociated with TB-A solution (in mM: 135 NaCl, 4 KCl, 1 MgCl_2_, 10 HEPES, 0.33 NaH_2_PO_4_, 5.5 glucose, 15 BDM, 5 mg/ml BSA, pH 7.2). The single isolated cardiomyocytes obtained from five mice per treatment were then seeded into ECM (Engelbreth-Holm-Swarm murine sarcoma, Sigma-Aldrich, cat. no. E1270-5ML) pre-coated 35 mm imaging Petri dish (Zell-Kontakt, cat no. 5160–168). In three steps of five minutes each, the Ca^2+^ concentration was increased from 0.24, 0.6 to 1.2 mM mixing TB-A and TB-B (in mM: 137 NaCl, 5.4 KCl, 1.8 CaCl_2_, 1 MgCl_2_, 10 HEPES, 5.5 Glucose, pH 7.4). The cardiomyocytes were kept in TB-B solution at 37 °C and 5% CO_2_ until use on the same day.

### Fluorescence dyes

The cell permanent Ca^2+^ fluorescence dye 10 µM Rhod-2-AM (Invitrogen, cat no. R1245MP) dissolved in TB-B was incubated for 45 min. For intracellular NO measurements, the cardiomyocytes were incubated with 10 µM trappable NO dye Cu_2_(FL2E) (Nitric Oxide Sensor Intracellular Kit “NO-ON”-FL2E, Strem Chemicals, cat no. 96-0396) for 2 h, as recommended by the manufacturer. Control solution and all drugs mentioned below were further dissolved in TB-B containing 200 µM l-Arginine (l-Arginine monohydrochloride, Sigma-Aldrich, cat no. A5131-10G). All other blockers and activators are summarized on Supplementary Table S8.

### Confocal microscopy and data acquisition

A confocal laser scanning microscope (Leica TCS MR 2) with a 63 × water immersion objective (PL Apo 63x/1.20 W CORR from Leica) was used. The NO fluorescence dye was excited by the Argon laser (488 nm) and the emission was detected by a photomultiplier in the spectral range of 497-537 nm. The Ca^2+^ fluorescence dye Rhod 2 was excited with the He/Ne laser (543 nm) and the emission light was detected by a photomultiplier between 551 and 701 nm. All images were saved as 8-bit images and had a pixel size of 0.186 µm and a time resolution of 800 lines per second. XYT images contained 512 × 512 pixels and XT images recorded 5632 lines with 512 pixels per line [27]. The line-scan trace was set at the center of the cardiomyocyte and away from the nucleus. Two seconds after the initial of the line scan, the cardiomyocytes were stimulated with a square pulse at 0.167 Hz, 20 V and 10 ms duration via two bath platinum electrodes connected to a stimulator (Stimulator, SI Heidelberg). Six line scans were recorded per cell with an interval of 6 s between each line scan. Other two frequencies of stimulations were given during tests of the protocol at 0.67 and 1 Hz. The images obtained from the NO-ON fluorescent dye were averaged and filtered offline by an unweighted moving average (*n* = 100) reducing noise, while the images from Rhod-2 were not filtered. For the analyses of the AUC, peak, delta-start parameters of the NO transient, each trace was normalized by the ratio between its maximal intensity (*F*_1_) over to its *F*_0_. The value of *F*_0_ was obtained from the average of 200 ms of the stable raw intensity signal recording just before the electrical stimulation (equivalent to 160 points; 1.25 ms/ point). The *F*_1_/*F*_0_ data were then analyzed with a custom-made code in Fiji and python3.5.2 (spyder, anaconda3) to calculate the physiological parameters showed on Table 1. Computer code is fully available upon request. Different calculation for the baseline parameter was used, strategy named as “normalized delta baseline (NDB)”. It was obtained subtracting the raw value of the first baseline from the raw value of the sixth baseline of the same cardiomyocyte and then divided by the highest raw value of the same group (NDB = Baseline1 − Baseline6)/maximum Baseline. The raw value was obtained from 200 ms before the electrical stimulation.

**Table 1:** Biophysical parameters of NO transients of WT and mdx-isolated cardiomyocytes recorded in the presence of the control solution

Genotype	WT (percentile 25, median, 75)			mdx (percentile 25, median, 75)		
Percentile	25	Median	75	25	Median	75
Biophysical parameter						
Baseline (a.u.)	17.07	22.50	28.43	14.33	19.43	37.23
Peak (*F*/*F*_0_)	0.072	0.131	0.230	0.076	0.130	0.196
Time to peak (ms)	114	131	161	108	130	159
Duration (ms)	316	430	553	305.6	455.6	645.9
Tau (ms)	154	216	278	143	230	323
Area under curve (*F*/*F*_0_*ms)	16.23	40.46	67.60	18.91	37.75	68.62
Peak NO/Ca^2+^ ratio	0.058	0.118	0.196	0.055	0.121	0.208
AUC NO/Ca^2+^ ratio	0.044	0.097	0.223	0.038	0.107	0.191
Δ peak Ca^2+^–NO (ms)	39.7	63.8	111.3	34.4	63.8	108.1
Δ start Ca^2+^–NO (ms)	46.3	67.5	98.8	42.5	63.1	89.0

### Consecutive Ca^2+^ transients

Cardiomyocytes obtained as described above from different set of five mice per treatment were used to record consecutive Ca^2+^ transients in a different set-up, as previously described [67, 111]. Isolated cardiomyocytes were incubated during 30 min with 5 µM Fluo-4-AM (Life Technologies, Carlsbad, CA, USA). Three 35 mm imaging Petri dishes were used for recording consecutive Ca^2+^ transients before and after different treatments. From each Petri dish, a minimum of 10 cardiomyocytes were recorded as controls and a minimum of 15 cells were recorded after drug application. Cardiomyocytes that did not respond the field stimulation with a single Ca^2+^ transient were discard from the analysis. The consecutive Ca^2+^ transients were recorded using an Olympus OSP-3 System, second Generation microscope connected to a Photomultiplier unit (Olympus, Tokyo, Japan) at 20X magnification equipped with a Xenon light source system, filtered to provide an excitation wavelength of 488 nm. A recording area of 7.5 µm^2^ and away from the nucleus was selected on the photomultiplier unit’s pinhole. Cardiomyocytes were flanked by a pair of platinum electrodes connected to a stimulator (Stimulator, SI Heidelberg) and single-twitch stimulations at 20 V during 10 ms at frequency of 2.0 Hz. TB-B containing 200 µM l-Arginine was used as control solution and to dissolve other drugs. Only cardiomyocytes responding to a single Ca transient upon electrical stimulation for 10 consecutive transients were selected and the fluorescence intensity were analyzed. Subsequently, TB-B was removed and 2 ml of the respective drug dissolved in TB-B was added into the petri dish. After a period of 10 min of incubation, consecutive Ca^2+^ transients were recorded as described above. The fluorescence intensity was sampled at 2 KB/s in a PC using LabChart Pro V8 (AD Instruments). The AUC calculated by Peak Area parameter from LabChart Pro V8′s Peak Analysis package of last six consecutive Ca^2+^ transients was normalized by the AUC of the fifth transient (100%).

Fluo-4 AM calibration procedure for converting the voltage signal into Ca^2+^ concentration was performed at the end of every experiment as previously described [67, 86, 108, 111]. Briefly, the background of the petri dish was recorded in 10 different spots (no cells). After incubation with calibration solution I containing (in mM): NaCl: 140; KCl: 5; KH_2_PO_4_: 1.2; MgCl_2_*6H_2_O: 1.2; CaCl_2_*2H_2_O: 4; HEPES: 20; Ionomycin: 0.005; CPA: 0.01; Caffeine: 5; Ouabain: 1 at room temperature, 10 cardiomyocytes incubated in 10 µM Fluo-4 AM at 37 °C for 30 min cells were recorded as maximum fluorescence (*F*_max_). Then, the Petri dish was washed twice with TB-B and incubated at room temperature with calibration solution II containing (in mM): LiCl: 140; KCl: 5; KH_2_PO_4_: 1.2; MgCl_2_*6H_2_O: 1.2; HEPES: 20; EGTA: 4; Ionomycin: 0.005; CPA: 0.01; Caffeine: 5; Ouabain: 1. At the end, 10 cells were recorded as minimum fluorescence (*F*_min_). The range of Ca^2+^ concentration to established *F*_max_ and *F*_min_ was 0 to 4 mM, respectively. Once both *F*_max_ and *F*_min_ were determined, the background was subtracted and then used to transform voltage into Ca^2+^ concentration using the following formula: [Ca^2+^]_i_ = Kd [(*F* − *F*_min_)/(*F*_max_ − *F*)] [33]. Kd is the apparent dissociation constant of the Fluo-4-AM provided by the manufacturer (345 nM) and *F* represents the Ca^2+^ transient signal in volts recorded directly from the photomultiplier.

### Western blotting

For each western blot, isolated cardiomyocytes obtained from five age-matched WT and five MDX were used. Proteins were extracted from murine cardiomyocytes using Whole Cell Lysis Buffer (WCLB) (in mM: 20 Tris–HCl, 150 NaCl, 1 Na_2_EDTA, 1 EGTA, 2.5 Na-Pyrophosphate, 1 Na-Vanadate, 1 PMSF, 1 DTT, 1% Triton, pH 7.5) and quantified using NanoDrop One using custom procedure at 290 nm wavelength. Protein separation was performed in 4–12% SDS-PAGE NuPAGE^®^ Gel (Invitrogen) for dystrophin and 10% for nNOS, iNOs, eNOS and GAPDH at 200 V for 50 min using 1X NuPAGE^®^ SDS Running Buffer for electrophoresis (Invitrogen; 50 mM MOPS, 50 mM Tris Base, 0.1% SDS, 1 mM EDTA, pH 7.7). Then, the separated proteins were transferred into PVDF-membrane with 1X NuPAGE^®^ Transfer Buffer (Invitrogen) with 20% methanol (in mM: 25 Bis–Tris, 25 Bicine, 1 EDTA, pH 7.2). Membranes were blocked with milk fat free 5% dissolved in 1X Tris-Buffer Solution (TBS; 100 mM Tris–HCl, 1.5 M NaCl (1.50 M) and pH 7.4 with NaOH) with 1% Tween 20 (TBS-T). The following primary antibodies diluted in TBS-T were incubated overnight at 4 °C: Dystrophin (Abcam, cat no. ab15277, 1:200); nNOS (Abcam, cat no. ab76067, 1:1000); iNOS (Abcam, cat no. ab15323, 1:400); eNOS (Abcam, cat no. ab50260, 1:1000); GAPDH (Abcam, cat no. ab181603, 1:10,000). The secondary antibody was diluted in TBS-T and incubated for one hour at room temperature: Goat Anti-Rabbit IgG (Abcam, cat no. ab205718, 1:20,000). Secondary antibodies were coupled to HRP (Horse Redish Peroxidase) allowing signal detection with the AceGlow Chemiluminescence Substrate (VWR International GmbH, cat no. 730-1511).

### Statistical analysis

Only cardiomyocytes responding to field stimulation with a single Ca^2+^-Transients were used for the statistical analysis. Statistically significant outliers were detected by the free online available GraphPad QuickCalcs (*α* = 0.05). Only those were removed from the data set and statistical analysis was performed with GraphPad Prism 7. For graphical presentation, boxplots were chosen where the line in the box represented the median and the 25% and 75% quartiles by the extremes of the box. The whiskers reached from the minimum to the maximum. The data sets were statistically compared by ANOVA with Bonferroni post hoc pairwise multiple comparisons vs. control. In each figure, the number of cardiomyocytes analyzed is represented by the letter n obtained from five mice of each genotype.

## Results

**Fig. 1:**
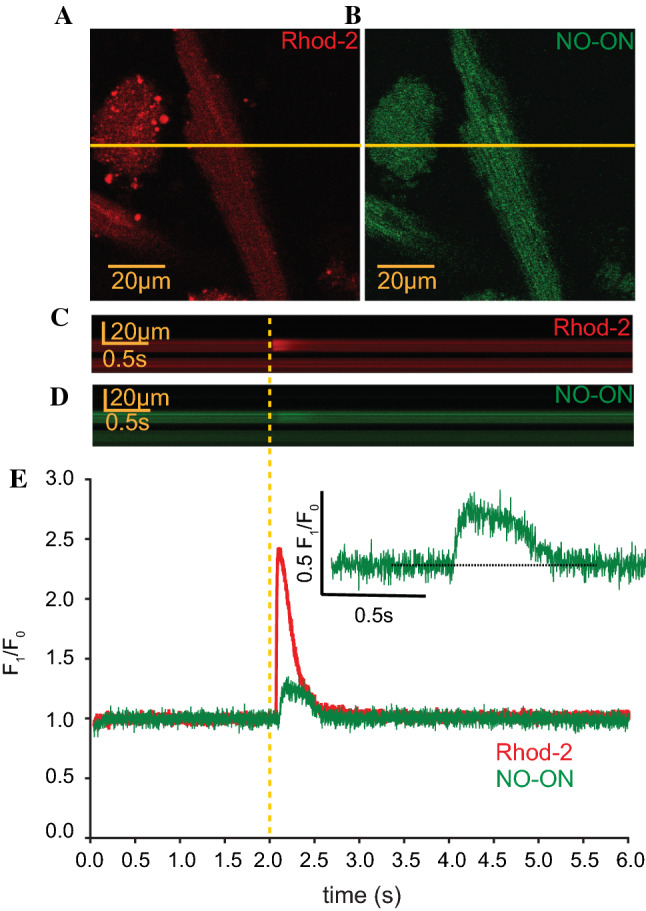
Fluorescence imaging of one isolated cardiomyocyte. An isolated cardiomyocyte loaded with Rhod-2 (10 µM for 45 min) **a** was excited with a laser wavelength of 543 nm, and emission was detected between 551 and 701 nm, while the cardiomyocyte loaded with NO-ON dye (10 µM for 2 h) **b** was excited at 488 nm, and emission was detected between 497 and 537 nm using a confocal laser scanning microscope (image size: 512 × 512 pixels, pixel size: 0.186 µm). The yellow line represents the line-scan region in the recorded cardiomyocyte. **c**, **d** Averaged image of six consecutive line scans (5632 lines with 512 pixels, 800 lines/s, 8 s of total line scanning) for simultaneous recordings of Ca^2+^ and NO transients along the yellow line from the cardiomyocyte shown in **a**, **b**. The dotted yellow line represents the moment at which a single electrical stimulation was given (20 V, 10 ms, 0.167 Hz) per line scan at 2 s after the initiation of recording and at another 6 s after the stimulation. **e** Normalized fluorescence intensity, *F*_1_/*F*_0_, of both the Ca^2+^ (red) and NO (green) traces of the averaged line scans in **c**, **d**. The time scale (s) is represented at the abscissa, and the ordinate represents the normalized fluorescence intensity *F*_1_/*F*_0_. The inlet shows a magnification of the NO transient from the trace represented in **e**. The dotted line shows that the baseline returned to the level before electrical stimulation. Individual traces of the six single line scans are shown in Fig. S1

Using confocal microscopy, line scans were recorded in cardiomyocytes isolated from 12-month-old WT mice that were double-loaded with 10 µM Rhod-2 for Ca^2+^ transient visualization (Fig. 1a) and 10 µM “NO-ON” for NO transients (Fig. 1b). Ca^2+^ transients were recorded as an internal control to evaluate (a) the viability of the cardiomyocyte, where only those that responded to each electrical stimulation with a single Ca^2+^ transient were selected for data analyses, and (b) to correlate the temporal NO production. At 2 s after the initiation of recording, a single squared electrical pulse (20 V amplitude, 10 ms duration) was applied to depolarize cardiomyocytes (dashed yellow line Fig. 1c–e). The recording continued for another 4 s to observe the NO production pattern related to electrical stimulation. From each cardiomyocyte isolated, six consecutive line scans were recorded (dashed yellow line Fig. 1c for Rhod-2 and Fig. 1d for NO-ON; non-normalized representative data Supplementary Fig. 1), normalized to their own baseline (*F*_1_/*F*_0_) and then averaged before being transformed into a representation *F*_1_/*F*_0_ trace as a function of time (Fig. 1e). Analysis with this method showed that both Rod-2 and NO-ON were stable for at least 36 s of recording, and a single transient was produced after the field electrical stimulation (Supplementary Fig. 1). Based on the established data and analysis criteria described above, no further significant variation in the NO line scan was observed before or after the NO-ON transient signal, which may have indicated the occurrence of another NO production event. Electrical stimulation-induced NO transients were also observed in cardiomyocytes loaded with only NO-ON fluorescent dye (non-normalized data from three different experiments in Supplementary Fig. 2), excluding the possibility of artifact signals from the Rhod-2 fluorescent dye or eventual cross-activation of the NO-ON dye by the other laser.

Ten different biophysical parameters were summarized to describe the NO transients (Table 1). The NO transients started approx. 67.5 ms after the beginning of the Ca^2+^ transient, with a median duration of 430 ms and a median peak of 0.131 *F*_1_/*F*_0_ (inlet Fig. 1e; Table 1). To quantify and thus understand the dimension of the NO transient observed herein, we correlated the ratios of the peak and area under the curve (AUC) values of the NO transient to the Ca^2+^ transient. This result showed that the NO median peak was 11.8% of the Ca^2+^ peak and that the NO median AUC was 9.7% of the Ca^2+^ median AUC (Table 1). No differences in the NO transient biophysical parameters were observed between WT and mdx-isolated cardiomyocytes (Table 1), suggesting that NO was evenly produced independent of the genotype.

**Fig. 2:**
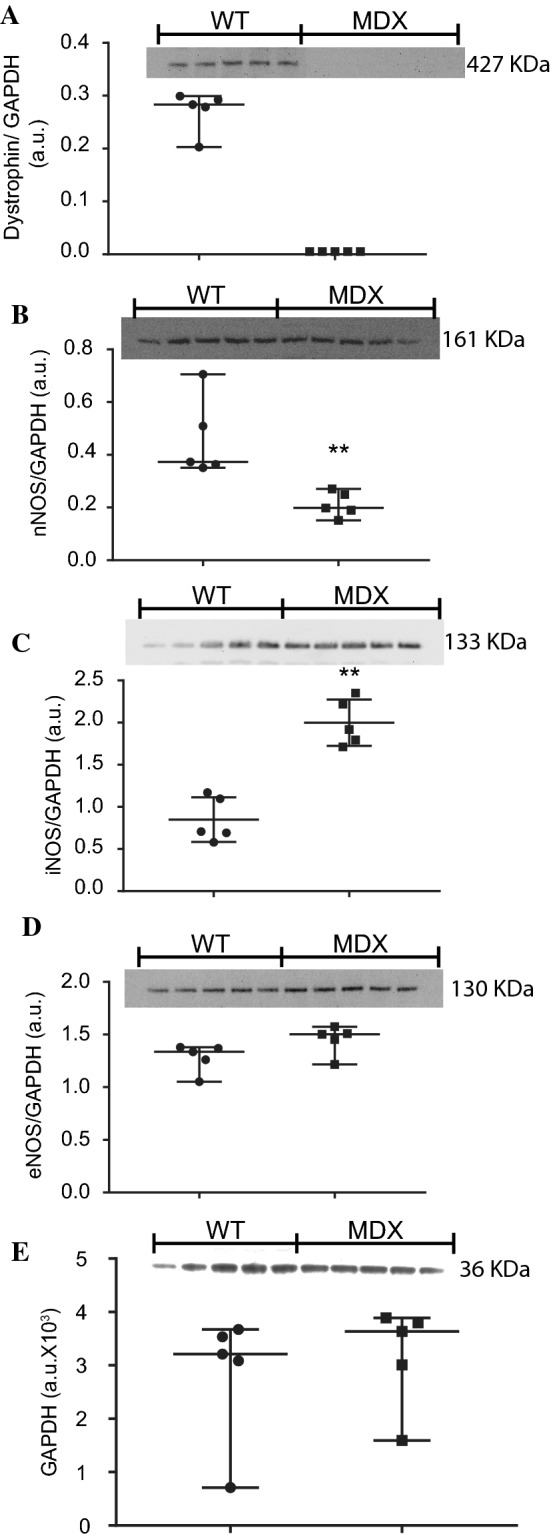
Immunoblotting of dystrophin and NOS in isolated cardiomyocytes. Proteins were extracted from cardiomyocytes isolated from five mouse hearts of each genotype. Seventy-five micrograms of protein per lane was used to perform immunoblot analysis of dystrophin (approx. 427 kDa) on a 4–12% gel (**a**), nNOS (approx. 161 kDa) on a 10% gel (**b**), iNOS (approx. 133 kDa) on a 10% gel (**c**), eNOS (approx. 130 kDa) on a 10% gel (**d**) and GAPDH (approx. 36 kDa) on a 10% gel (**e**). Nonparametric statistical analyses were performed on the normalized intensity signals, and the raw intensity of GAPDH was used as the loading control (**e**). **p < 0.01. *ns* no significant difference

Next, we investigated the differential protein expression of dystrophin (Fig. 2a), nNOS (Fig. 2b), iNOS (Fig. 2c) and eNOS (Fig. 2d) on isolated cardiomyocytes by immunoblot following the same protocol as that used to record the NO and Ca^2+^ transients from five mice of each genotype (full membrane Supplementary Fig. 3). All values were normalized to the signal intensity of GAPDH (Fig. 2e), and the raw intensities did not differ statistically between the genotypes. As predicted, the expression of dystrophin was present in WT cardiomyocytes and absent in mdx cardiomyocytes. The protein expression of nNOS was significantly reduced in mdx cardiomyocytes compared to WT cardiomyocytes (Fig. 2b), while iNOS expression was significantly increased (Fig. 2c). The expression of eNOS was not significantly different between the two types (Fig. 2d).

**Fig. 3:**
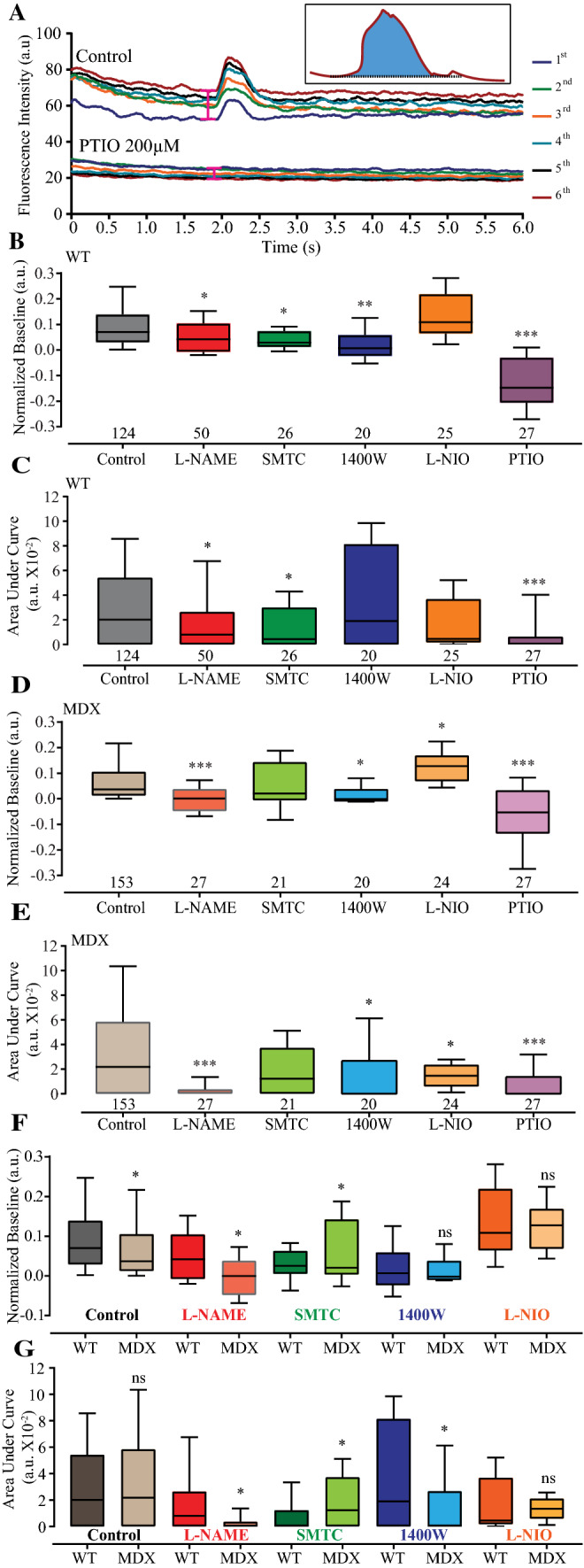
Baseline and AUC values of endogenous NO production. Representative recording of six consecutive NO transients (first–sixth) from cardiomyocytes isolated from 12-month-old WT mice and electrically stimulated (20 V, 10 ms) in a control solution (containing l-arginine 200 µM) and under treatment with the NO scavenger PTIO (200 µM) (**a**). The pink range symbol at the traces and before 2.0 s represents the approximate region of 200 ms used to analyze the baseline. The inlet shows the analyzed area under the curve (AUC) of the sixth NO transient in blue. All six traces (first to sixth) are color-coded and identified on the right side of the traces. The fluorescence intensity is shown in arbitrary units at the ordinate, and the time is shown in s at the abscissa. **b**–**e** Boxplots of the normalized baseline (**b**, **d**) and AUC (**c**, **e**) values illustrated on the ordinate in median arbitrary units (m.a.u.) from the NO transients from WT (**b**, **c**) and mdx (**d**, **e**) cardiomyocytes. Baseline (**f**) and AUC (**g**) comparisons between genotypes. Isolated cardiomyocytes under the effects of different pharmacological agents dissolved in control solution containing 200 µM l-arginine (abscissa): control (gray), 5 mM L-NAME (red), 100 nM SMTC (green), 1 µM 1400 W (blue), 1 µM L-NIO (orange) and 200 µM PTIO (purple). The numbers of analyzed cardiomyocytes per treatment obtained from five mice per genotype are shown under the boxplots. Comparison vs. the respective control group: *p < 0.05; **p < 0.01; ***p < 0.001. For the exact data values, see Table S1 for the normalized baseline values and Table S2 for the AUC values

These results suggested that, according to the pathophysiological condition, the activities of the differentially expressed NOS isoforms were responsible for producing NO during diastole (named baseline) and systole (named NO transient). To test this hypothesis, six consecutive line scans were recorded, normalized to the baseline (*F*_1_/*F*_0_) and then averaged with other recordings according to the genotype and pharmacological tool using the same experimental set-up and data analyses as those described in Fig. 1. As seen on representative raw NO recordings (i.e., before normalization), no significant alteration in the NO-ON fluorescence signal was observed prior to electrical stimulation (from 0 to 2.0 s) or after the NO transient, which lasted 4 s after stimulation (Fig. 3a). However, it was interesting to observe that the baseline of the NO signal systematically increased after each stimulation (analyzed at the pink marker before 2.0 s as shown in Fig. 3a), suggesting increasing or continuous NO production during diastole (baseline) after each stimulation (Fig. 3a). In comparison to that of the control solution, the baseline of WT-isolated cardiomyocytes from five mice was significantly reduced by the nonspecific NOS blocker *N*-nitro-l-arginine methyl ester (L-NAME, 5 mM), as well as by the specific nNOS blocker *S*-methyl-l-thiocitrulline (SMTC, IC_50_ 100 nM) [48] and the specific iNOS blocker 1400 W (IC_50_ 1 µM) [73]. However, it was not reduced by the eNOS blocker l-iminoethyl ornithine (L-NIO, IC_50_ 1 µM) [22] (Fig. 3b; Table S1). The NO scavenger 2-(4-carboxyphenyl)-4,4,5,5-tetramethylimidazoline-1-oxyl-3-oxide (carboxy-PTIO, PTIO hereafter) at 200 µM [40] was used to exclude the possibility of artifact signals produced by byproducts or cytotoxicity of the NO-ON fluorescent dye. It is relevant to note that the presence of PTIO not only discarded the possibility of cytotoxicity artifacts caused by the NO-ON fluorescent dye or the laser but also significantly reduced the NO baseline signal produced in comparison to that of the control, supporting previous observations of continuous NO production during diastole. Combining the data analysis from control and PTIO-treated cardiomyocytes, we ruled out artifact signal acquisition from NO-ON due to cytotoxicity because PTIO could reduce the NO-ON signal, and no NO-ON bleaching occurred because the baseline signal increased without PTIO.

The isoform responsible for the NO transient was determined using a low concentration of the specific isoform of the NOS blocker and analyzing the integration of the amplitude over time, as shown by the blue area on the inlet in Fig. 3a, hereafter named the AUC. Figure 3c summarizes the data analyses in comparison to the control condition, where both L-NAME and SMTC significantly reduced the AUC of the NO transient. Neither the iNOS blocker 1400 W nor the eNOS blocker L-NIO (Table S2) significantly reduced the NO transient AUC parameter. However, the AUC of the NO transient was effectively reduced in three independent experiments using PTIO. These results suggested that in isolated WT cardiomyocytes, the NO transient is nNOS-derived. It is important to note that under control conditions, 23.3% (29 out of 124) of the recorded and analyzed isolated cardiomyocytes did not produce NO transients upon electrical stimulation.

The normalized delta baseline and AUC values of the NO transients analyzed in mdx cardiomyocytes were similar to those obtained from WT cardiomyocytes (Fig. 3d, e; Table S1). In comparison to the control solution, L-NAME, 1400 W and PTIO significantly reduced the baseline recorded from mdx cardiomyocytes (Fig. 3d; Table S2). Interestingly, the presence of L-NIO significantly increased the both raw and normalized delta baseline NO production. Treatment with L-NAME, 1400 W, L-NIO or PTIO significantly reduced the NO transient AUC parameters in dystrophic cardiomyocytes (Fig. 3d; Table S2). As expected, SMTC did not affect the baseline or the AUC of NO transients of dystrophic cardiomyocytes. Taken together, these data suggested that both the iNOS and eNOS isoforms are responsible for the NO transient in dystrophic cardiomyocytes. Similar to WT cardiomyocytes, 24.2% (37 out of 153) of the dystrophic cardiomyocytes showed Ca^2+^ transients without NO transients.

When comparing NO production differences between normal and dystrophic cardiomyocytes, we observed that unlike in WT cardiomyocytes, the NO baseline production in mdx cardiomyocytes was significantly reduced by both the control solution and L-NAME treatment and increased by SMTC. There were no genotype differences between the 1400 W and L-NIO treatment groups (Fig. 3f). The significant decrease in iNOS-derived NO baseline production in both WT and mdx cardiomyocytes suggested that iNOS is the isoform responsible for diastolic NO production. A similar analysis was performed for the NO transient by measuring the AUC (Fig. 3g). After electrical stimulation, there was no difference between WT and mdx cardiomyocytes; however, L-NAME and 1400 W induced significant reductions in the NO transient in mdx cardiomyocytes. SMTC treatment had the opposite effect, as the NO transient was significantly reduced in WT cardiomyocytes in comparison to mdx cardiomyocytes (Fig. 3g). Finally, L-NIO induced no differences in the NO signals between the two groups (Fig. 3g). This analysis supports the initial perception that NOS-derived NO transient production was differentially produced according to the pathophysiological characteristics of the cardiomyocytes.

Knowing that reactive oxygen species (ROS) induces NO production [84, 85], here it was evaluated whether ROS would form a transient signal of NO by incubating WT cardiomyocytes isolated from four mice with 150 μM H_2_O_2_ for 5 min (Supplementary Fig. 4). Under this specific condition, significant changes in the baseline NO or NO transient production were not observed, unlike after electrical stimulation. This result suggested that NO-dependent ROS production requires a longer time and most likely a different mechanism from that of electrical stimulated-derived NO transient signals.

**Fig. 4:**
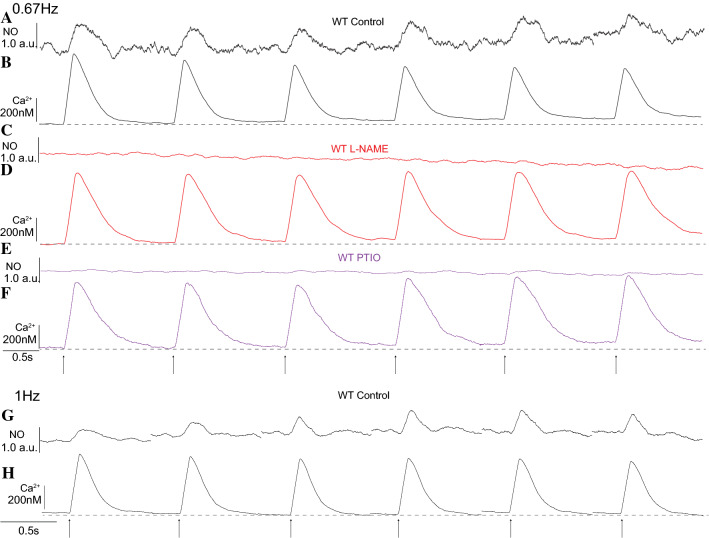
Last six consecutive NO and Ca^2+^ transients from WT cardiomyocytes. Representative traces from six non-normalized and consecutive NO transients in arbitrary units (**a**, **c**, **e**, **g**) and Ca^2+^ transients in nM (**b**, **d**, **f**, **h**) recorded from WT-isolated cardiomyocytes. **a**, **g** NO transient and **b**, **h** Ca^2+^ transient signals recorded in the control solution. **c** NO transient signal and **d** Ca^2+^ transient signals recorded from WT-isolated cardiomyocytes incubated with the nonspecific NOS blocker L-NAME (5 mM). **e** NO transient and **f** Ca^2+^ transient signals recorded from WT-isolated cardiomyocytes incubated with the NO scavenger PTIO (100 µM). The dashed gray line underneath the Ca^2+^ transient traces serves as a visual reference for the baseline. Each transient was triggered with 20 V, 10 ms, and 0.67 Hz (**a**–**f**) and 1 Hz (**g**, **h**) field electrical stimulations as indicated at the bottom with black arrows

**Fig. 5:**
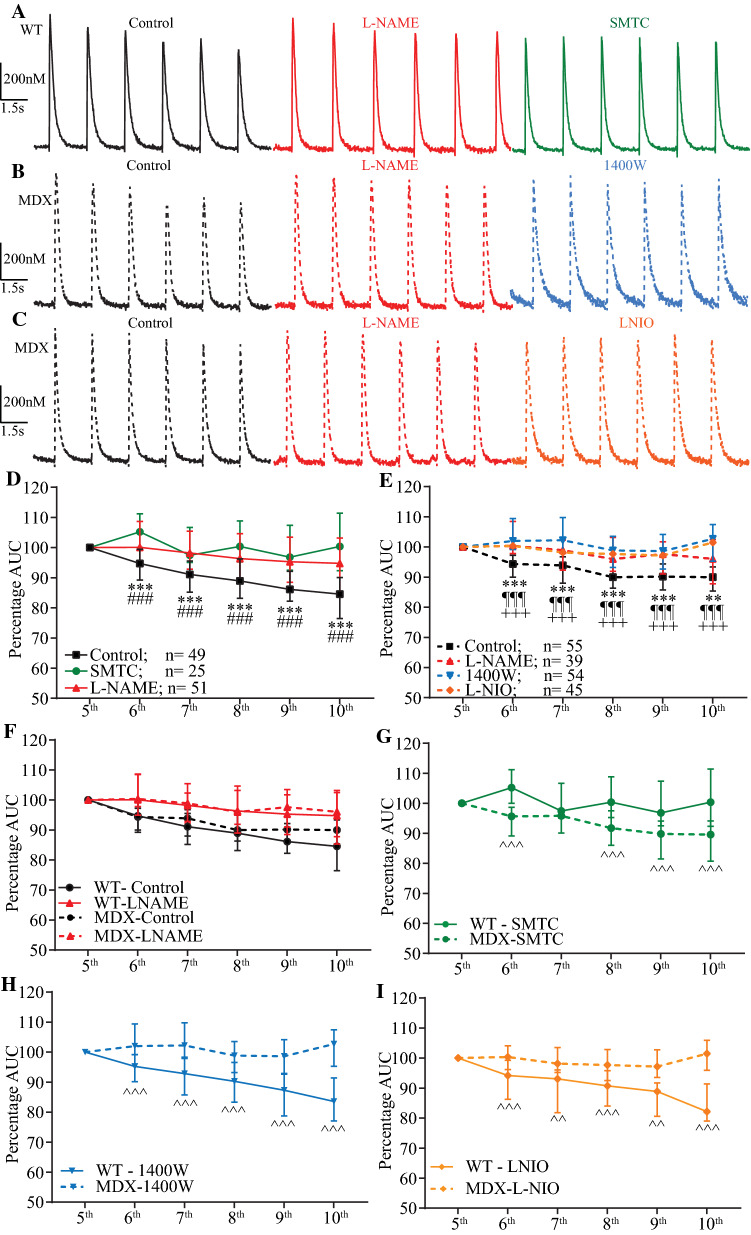
Effect of endogenous NO on consecutive Ca^2+^ transients. The last 6 of 10 calibrated and consecutive Ca^2+^ transients under the effect of specific blockers of each NOS isoform. Isolated cardiomyocytes were loaded with 10 µM Fluo4-AM, electrically stimulated (20 V, 10 ms, 2.0 Hz) and recorded at 2 Kb/s from the photomultiplier-amplified signal. The scale of the Ca^2+^ transient concentration (20 nM) is shown at the ordinate, and time (1.5 s) is shown at the abscissa. **a** WT-isolated cardiomyocyte Ca^2+^ transients recorded in the presence of the control solution (black), 5 mM L-NAME (red) and 100 nM SMTC (green) are shown. Consecutive Ca^2+^ transients from mdx cardiomyocytes in the presence of the control solution containing 200 µM l-arginine (dotted black) or different pharmacological agents dissolved in control solution containing 200 µM l-arginine (abscissa) are shown as follows: 5 mM L-NAME (dotted red) and 1 µM 1400 W (dotted blue) (**b**) and control solution (dotted black), 5 mM L-NAME (dotted red) and 1 µM L-NIO (dotted orange) (**c**). **d**, **e** Quantification of the percentages of the AUCs on the abscissa and the last five consecutive Ca^2+^ transients at the ordinate for the WT cardiomyocytes in **d** and the mdx cardiomyocytes in **e**. The fifth Ca^2+^ transient was considered the first Ca^2+^ transient to be analyzed and thus accounted for 100% of the AUC. The numbers of cardiomyocytes analyzed per treatment obtained from five mice per genotype are shown next to the legend. Comparison vs. the respective control transient: L-NAME **p < 0.01 and ***p < 0.001; SMTC ^¶¶¶^*p* < 0.001; 1400 W ^###^*p* < 0.001; L-NIO ^+++^*p* < 0.001. For the exact data values, see Table S3

The physiological implication of this brief endogenous NO transient on the well-established modulatory effect of NO on Ca^2+^ transients was investigated based on an exploratory study on different data sets of isolated cardiomyocytes reloaded with NO-ON and Rhod-2. In this study, the last 6 of 10 consecutive NO and Ca^2+^ non-normalized transients recorded were analyzed at two different frequencies of stimulation (0.67 Hz and 1 Hz). Each line scan is separated from the next one to indicate a new line scan and the new baseline after each stimulation. The Ca^2+^ transient was reduced after each stimulation in the control solution (Fig. 4a, b, g, h), but this reduction was absent in cardiomyocytes treated with L-NAME (Fig. 4c, d) or PTIO (Fig. 4e, f). In the control solution and after 0.67 Hz of stimulation, the NO baseline production increased after each stimulation, supporting our previous result regarding diastolic NO production. This preliminary result supports previous data [21] and further suggests that electrically triggered NO transients modulate Ca^2+^ transients independently of the stimulation frequency. Based on these exploratory results, we further analyzed and quantified the endogenous NO effect on the last 6 of 10 AUC Ca^2+^ transients obtained from electrically stimulated (20 V, 10 ms, 2.0 Hz) age-matched WT and mdx-isolated cardiomyocytes loaded with only 10 µM Fluo4-AM using a faster data acquisition set-up. Higher frequency of stimulation was chosen to avoid large Ca^2+^ transients due to inactivation. In comparison to the control solution, L-NAME and the other specific blockers used herein had no significant effects on the measured intracellular diastolic Ca^2+^ (baseline) from both WT and mdx cardiomyocytes. However, in isolated WT cardiomyocytes, the reduction of the last 6 consecutive AUC Ca^2+^ transients observed in the control solution was prevented with L-NAME and the specific nNOS blocker SMTC (Fig. 5a) but not with the iNOS blocker 1400 W or the eNOS blocker L-NIO (Supplementary Fig. 5). Two-way ANOVA indicated a significant interaction effect between NOS blockers and consecutive transients (Table S3), as the percentage of the WT AUC Ca^2+^ transients under L-NAME or SMTC treatment was significantly larger than that under treatment with the control solution (Fig. 5d). Dystrophic cardiomyocytes did not reduce the percentage of AUC Ca^2+^ transients upon consecutive stimulations when treated with L-NAME, 1400 W or L-NIO (Fig. 5b, c). Two-way ANOVA reported a significant interaction effect between NOS blockers and consecutive transients (Table S3), as the percentage of dystrophic AUC Ca^2+^ transients under L-NAME, 1400 W or L-NIO treatment was significantly larger than that achieved with the control solution (Fig. 5e).

The AUC of the Ca^2+^ transient is determined by peak and duration parameters, which were analyzed to investigate whether one or both would be affected by the NO transient. In WT-isolated cardiomyocytes, the patterns of peak and duration were similar to the AUC analysis. Two-way ANOVA of peak parameter showed a significant interaction effect between NOS blockers and consecutive transients (Table S4), as the percentage of the WT AUC Ca^2+^ transients under L-NAME or SMTC treatment was significantly larger than that under treatment with the control solution (Supplementary Fig. 7a). Although the pattern of the duration response was also similar to AUC, where L-NAME and SMTC were significantly higher in most of the transients, two-way ANOVA indicated no significant interaction between the number of the transient and NOS blockers (Supplementary Fig. 7b; Table S5). As expected from the results of the AUC, 1400 W and L-NIO did not show any difference in comparison to control (Supplementary Fig. 7a, b).

Similar analyses of peak and duration parameters were performed using dystrophic cardiomyocytes. Two-way ANOVA indicated significant interaction between consecutive transient and NOS blockers and in comparison to control; peak parameter was significantly higher in presence of L-NAME, 1400 W or L-NIO (Supplementary Fig. 7c; Table S4). SMTC did not change the peak of the Ca^2+^ transient. It is interesting to note that the treatment of all blockers used here did not modify the duration of the Ca^2+^ transient from dystrophic cardiomyocytes (Supplementary Fig. 7d; Table S5). These results suggest that from two parameters that define the AUC, the peak is the one affected in both WT and MDX genotypes, while with no relevance regarding the duration of the Ca^2+^ transient in dystrophic cardiomyocytes.

**Fig. 6:**
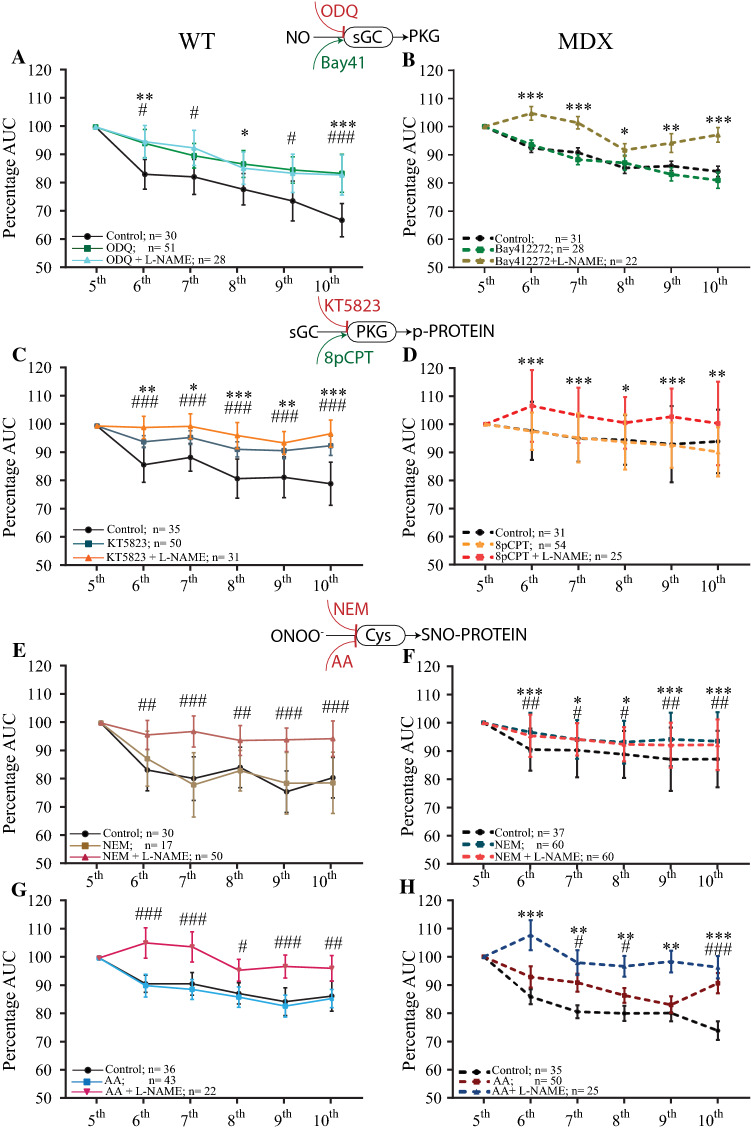
Quantification of the AUCs of the last six consecutive Ca^2+^ transients from WT and mdx cardiomyocytes in the presence of specific activators or blockers of NO signaling pathways. Isolated cardiomyocytes were loaded with 10 µM Fluo4-AM, electrically stimulated (20 V, 10 ms, 2.0 Hz) and recorded at 2 Kb/s from the photomultiplier-amplified signal. The data are in comparison to the control solution (black) in reference to the point on the NO signaling pathway indicated between panels. **a**, **c**, **e**, **g** Quantification of AUCs from WT-isolated cardiomyocytes and **b**, **d**, **f**, **h** mdx-isolated cardiomyocytes. **a** Effects of the sGC blocker ODQ (10 µM, red) and the combination of ODQ and 5 mM L-NAME (green) in comparison to the control solution (black). **b** Effects of 10 µM BAY-412272 (green) and the combination of BAY-412272 and 5 mM L-NAME (brown). **c** Effects of the PKG blocker KT5823 (10 µM, petrol) and the combination of KT5823 and 5 mM L-NAME (orange). **d** Effects of the PKG activator 8pCPT (10 µM, orange) and the combination of 8pCPT and 5 mM L-NAME (red). **e** Effects of the *S*-nitrosylation scavenger NEM (20 µM, brown) and the combination of NEM and 5 mM L-NAME in WT cardiomyocytes. **f** Effects of the *S*-nitrosylation scavenger NEM (20 µM, petrol) and the combination of NEM with 5 mM L-NAME (red) in mdx cardiomyocytes. **g**, **h** Effects of 1 mM ascorbic acid (AA) on *S*-nitrosylation (gray) and in combination with 5 mM L-NAME (dark red) in WT cardiomyocytes. **h** Effects of 1 mM AA on S-nitrosylation (dark blue) and in combination with 5 mM L-NAME (dark red) in mdx cardiomyocytes. The numbers of cardiomyocytes analyzed per treatment obtained from five mice per genotype are shown next to the legend. The symbols *, ** and *** represent *p* < 0.05, *p* < 0.01 and *p* < 0.001, respectively, of the single treatment in comparison to the control. The symbols #, ## and ### represent *p* < 0.05, *p* < 0.01 and *p* < 0.001, respectively, of the combined treatment in comparison to the control

Next, we reasoned that according to the cardiomyocyte pathophysiological condition, different NO signaling pathways would be activated, reducing the AUC of the NO-dependent Ca^2+^ transient. Again, we used pharmacological tools to block or activate specific points on the NO-sGC-PKG or NO-SNO signaling pathway instead of mutants to observe an acute effect and to avoid compensatory activities of other NOS isoforms [6, 92, 95, 109]. In comparison to the control solution, the sGC blocker 1H-[1,2,4]oxadiazolo[4,3,–a]quinoxalin-1-one (ODQ, 10 μM) had an effect similar to that of L-NAME (5 mM), as no significant reductions in the AUCs of the last six Ca^2+^ transients from three WT mice were observed (Fig. 6a; Table S6). Bay41-2272, an activator of sGC, had no significant effect on dystrophic cardiomyocytes in comparison to that of the control solution, and the effects of both the control and Bay41-2272 treatments were significantly reduced in comparison to those of L-NAME (Fig. 6b; Table S7). No significant effects on the AUCs of the last six Ca^2+^ transients under ODQ treatment were observed in dystrophic cardiomyocytes or under Bay41-2272 treatment in WT cardiomyocytes (Table S6). At the level of PKG activity, the blocker KT5823 (1 μM) significantly increased the Ca^2+^ AUC in WT cardiomyocytes in comparison to that achieved with the control solution, as detected for L-NAME (Fig. 6c). The effect of the PKG activator 8pCPT (10 μM) was similar to that of the control solution in mdx cardiomyocytes and was significantly reduced in comparison to that of L-NAME (Fig. 6d). The 8pCPT agent had no significant effect on WT cardiomyocytes, and KT5823 had no significant effect on dystrophic cardiomyocytes (Tables S6, S7). Both reductants of S-NO, N-ethylmaleimide (NEM, 20 μM) and ascorbic acid (AA, 1 mM) by themselves had no effects on the AUC of Ca^2+^ transients from WT cardiomyocytes. In comparison to the control solution, NEM and AA significantly increased the AUC only in the presence of L-NAME (Fig. 6e). However, in mdx cardiomyocytes, NEM or AA alone did significantly increase the AUC from the consecutively recorded Ca^2+^ transients, similar to the effect of L-NAME alone.

Since AA is also a scavenger of reactive oxygen species (ROS), we decided to test whether ROS is involved in the reduction of Ca^2+^ transients using 10 mM N-acetylcysteine (NAC), a specific ROS scavenger that is widely used in cardiomyocytes [102, 103] and skeletal muscle [14]. The Ca^2+^ transients in NAC-treated WT cardiomyocytes did not differ from those of the control, but differences were observed when NAC was used in combination with L-NAME (Supplementary Fig. 6A). An analogous approach was used with dystrophic cardiomyocytes, yielding results similar to those obtained with AA (Supplementary Fig. 6B), where only the 10th transient of NAC-treated cardiomyocytes was significantly different from that of the control. The mdx cardiomyocytes treated with NAC and L-NAME in combination showed a result similar to that achieved with L-NAME alone, where all Ca^2+^ transients were significantly different from those of the control. These results suggested that the WT cardiomyocytes were probably not influenced by ROS, while the main effect observed for mdx cardiomyocytes derived from NO with some contributions by ROS.

## Discussion

**Fig. 7:**
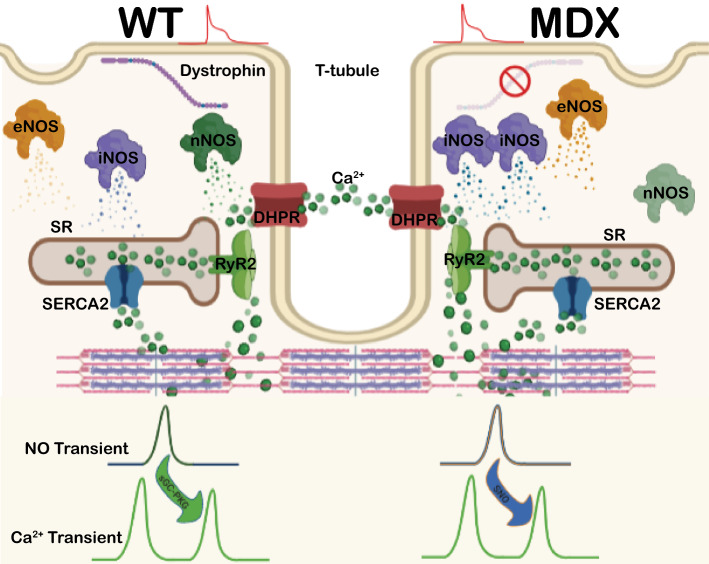
Schematic representation of the NOS-dependent NO transient and its effect on the Ca^2+^ transient. In normal cardiomyocytes (left side; WT) in which dystrophin is present, nNOS and iNOS are responsible for diastolic NO production, and nNOS is responsible for the systolic NO transient. The NO transient is produced after membrane depolarization (red), and it exerts its effect by reducing the next Ca^2+^ transient via the sGC-PKG signaling pathway. In dystrophic cardiomyocytes (right side; mdx), the absence of dystrophin reduces the expression and activity of nNOS and enhances iNOS, thus regulating diastolic NO production. During systole, iNOS and eNOS are responsible for the NO transient modification of the next Ca^2+^ transient via SNO modifications

A novel physiological paradigm for intracellular NO production is summarized in Fig. 7, proposing that endogenous NOS-dependent NO is transiently produced after the Ca^2+^ transient upon electrical stimulation. The NO production uncovered in this study is suggested to reduce the next Ca^2+^ transient via a signaling pathway-dependent mechanism according to the pathophysiological condition. In WT cardiomyocytes, the NO transient was nNOS-dependent, while it was eNOS- and iNOS-derived in mdx cardiomyocytes. In the WT group, the endogenous NO transient effectively reduced the subsequent Ca^2+^ transient via the NO-sGC-PKG signaling pathway, while this reduction was SNO-mediated in dystrophic cardiomyocytes. Another novelty observed herein is the roles of nNOS and iNOS in WT cardiomyocytes and iNOS and eNOS in mdx cardiomyocytes in NO production during diastole. Interestingly, in mdx cardiomyocytes, L-NIO significantly increased the baseline, suggesting a compensatory increase in iNOS activity once eNOS activity was blocked. Therefore, the NO transient in isolated murine cardiomyocytes exerts a negative modulatory effect on the subsequent Ca^2+^ transient during the ECC.

### NO is transiently produced in cardiomyocytes

Since this was the first attempt to record intracellular NO production in real time, we analyzed select cardiomyocytes at 2 s before and 4 s after electrical stimulation. Three conditions were established to analyze the recorded data. First, we avoided shape variance and movement during stimulation using 2,3-butanedione monoxime (BDM) during cell dissociation and checking for movement artifacts on line-scan traces. Second, since NOS-derived NO is directly (nNOS and eNOS) and indirectly (iNOS throughout activation of contraction-dependent signaling pathway) Ca^2+^ release and only a single Ca^2+^ transient is triggered by electrical stimulation, we use it as a well-established physiological reference event produced in cardiomyocytes [32, 51] to evaluate NO production during or after the ECC. Finally, the NO-ON dye signal was present only after the initial Ca^2+^ transient. The resulting NO transient lasted for approx. 430 ms during the Ca^2+^ transient and the AUC of the NO transient was only 11% of that of the Ca^2+^ transient. Similar transient production of NO was previously reported extracellularly in rat and rabbit hearts using porphyrinic chronoamperometric microsensors covered with Nafion [76]. In situ, extracellular NO transients are recorded after systole and blocked by L-NMMA, a nonspecific NOS blocker [76]. In isolated cardiomyocytes, extracellular NO is detected after application of an external mechanical force to the cell membrane. Although this technique allows for the rapid detection of extracellular NO fluctuations, the disadvantages lie in not knowing the amount of intracellular NO produced and the concentration decreasing proportionally to the square of the distance between the target cell and microelectrode. For instance, Pinsky et al. could detect NO transient production only by placing the porphyrinic microelectrode 2–3 μm from the cell membrane and were unable to detect any signal at a distance of 35 μm [76]. This well-known issue of the chronoamperometric technique was addressed in this study, as we showed that the initial NO transient occurred during systole using a sensitive fluorescent dye. Here, we precisely report the activation of intracellular NO production after the membrane depolarization-induced Ca transient.

It is interesting to note that intracellular NO transients were produced under different frequencies of stimulation (0.167, 0.67 and 1.0 Hz), suggesting a physiological process that is capable of adapting to different heart frequencies. Furthermore, the subsequent baseline was increased, suggesting constant NO basal production after electrical stimulation, despite the normal bleaching process of any fluorescent dye recorded under low amplitude scale as observed in Fig. 3. To discard the possibilities of recording and analysis artifacts, such as phototoxicity from the NO-ON dye, the NO scavenger PTIO was used, and no significant differences in the baseline signals were observed between the first and sixth stimulation events. Therefore, the results from the control and PTIO-treated protocols suggest that no artifacts of NO-ON signal recording derived from cytotoxicity occurred because (1) of the specificity of the NO-ON fluorescent dye [62–64] and (2) the fact that PTIO was able to reduce the NO-ON signal, supporting the evidence that the NO-ON dye is specific for NO [62, 63]. We also ruled out the possibility of significant bleaching or bleeding of the NO-ON dye because the baseline signal increased after each stimulation in control (without PTIO) cardiomyocytes. According to the genotype, the source (isoform) of diastolic NO production varied, suggesting that all three isoforms contribute to basal NO production. In comparison to WT cardiomyocytes, dystrophic cardiomyocytes produced significantly lower amounts of NO during diastole, supporting previous reports of mdx cardiomyocytes producing lower levels of NO [23, 82]. Interestingly, there was no difference in NO production during diastole between WT and mdx cardiomyocytes, as both genotypes exhibited reduced NO production after treatment with 1400 W and increased NO production after treatment with L-NIO. This analysis suggests that the activity of the NOS isoform dictates a compensatory mechanism, as previously described for the heart [95] and other tissues [31, 41, 94].

We noticed that approx. 24% of the recorded cardiomyocytes did not produce NO signal despite always presenting normal Ca^2+^ transients, and these cardiomyocytes without NO signals were present in all plates and in between cardiomyocytes with successful NO transients. To eliminate a possible technical artifact (loading), we attempted to record the NO signals more than once at different time points in the cardiomyocytes. To exclude the possibility of a low loading capacity, we increased the intensity of the laser to record a small NO signal, but no transient signal was successfully recorded. It is well established that anatomical and physiological regional differences exist in the ventricle, such as differences in wall thickness; Na^+^, K^+^ and Ca^2+^ currents; and action potentials [3, 17, 58, 61]. Porphyrinic microsensors revealed differential NO production according to the placement of the probe in the heart, where the endocardium produced more NO than the myocardium [76]. Similarly, heterogenic NOS isoform expression in WT tissue has been reported across different layers of the right and left ventricles, where the nNOS and iNOS expression patterns are opposite that of eNOS [12, 13]. Therefore, these data collectively suggest that due to the heterogeneity of ventricular tissues, cardiomyocytes from the left ventricular epicardium and right ventricle expressing lower amounts of nNOS and iNOS do not produce NO transients after electrical stimulation.

### Differential NO sources are dependent on NOS isoform expression

Two different approaches were used with the aim of identifying different NOS isoforms that could be responsible for the NO transient. First, immunoblot analyses of isolated cardiomyocytes were performed, showing that the three isoforms were differentially expressed in WT and mdx cardiomyocytes. The protein level of the nNOS isoform was significantly reduced in mdx cardiomyocytes compared to that in WT cardiomyocytes, while that of iNOS was significantly enhanced, and the eNOS protein expression was not significantly different between the two groups. Our results support previous reports demonstrating that cardiomyocytes express nNOS [90], specifically in the sarcoplasmic reticulum [18, 109] and at the sarcoplasm membrane [104]. eNOS is detectable in spatial microdomains of plasma membrane, such as caveolae and lipid rafts and at the sarcoplasmic membrane [25, 59, 115], and iNOS is normally expressed under physiological conditions in the cardiomyocyte cytoplasm, perinuclear space, Golgi complex, contractile fibers, but not in the sarcoplasmic reticulum [4, 5, 10–12, 57, 90]. The second approach was using pharmacological agents at the IC_50_ and thus specifically inhibiting only one NOS isoform. In WT cardiomyocytes, the AUC of the NO transient was significantly reduced in the presence of L-NAME and SMTC [49], while no significant alterations were observed in the presence of 1400 W [73] or L-NIO [22]. In contrast, the AUC of the NO transient in mdx mice was significantly reduced by L-NAME, 1400 W or L-NIO. This evidence suggests that WT and mdx cardiomyocytes produce NO transients utilizing different isoforms. Moreover, the presence of the NO scavenger PTIO on both genotypes significantly abolished the NO transient, supporting the data obtained from the specific blockers. Together, these results suggested that differential NOS isoform expression determines the NO transient source, thus resulting in NO production by a different isoform according to the pathophysiological condition. In WT and mdx mice of an age comparable to those used in our immunoblot and NO transient experiments (12 months old), Bia et al. showed no difference in total NOS activity in the myocardia of the two groups. However, similar to what we observed, the nNOS activity was significantly reduced in mdx cardiomyocytes, while the iNOS activity was significantly enhanced, and no difference in the activity of eNOS was observed [11]. Cardiomyocytes treated with 7-NINA, another specific nNOS blocker, showed a reduction in NO production to approx. 75% of that in the WT, with no difference being observed in mdx cardiomyocytes, indicating that NO is mainly derived from an isoform other than nNOS in mdx mice [82]. Therefore, in combination with previous data [11, 76, 82], our results support previous evidence that murine cardiomyocytes transiently produce NO in an isoform-dependent manner, extending as a possible compensatory activity in mdx cardiomyocytes from iNOS and eNOS in response to the reduced nNOS activity.

The characterization of NO production was also extended to the baseline, as the baseline production also decreased after the electrical stimulation of WT cardiomyocytes treated with 1400 W. This result was supported and extended by the data obtained with PTIO, showing that NO was produced not only after electrical stimulation and then followed by the Ca^2+^ transient but also constantly at low levels. Therefore, these results suggest that iNOS is the Ca^2+^-independent isoform responsible for diastolic NO baseline production and that at least two NO production events occur: one transiently during systole after the sarcoplasmic membrane depolarization-induced Ca^2+^ transient and another constantly during diastole.

### The NOS-dependent NO transient reduces the next Ca^2+^ transient

The role of each NOS isoform regarding the general modulatory effect of NO on the ECC and on Ca^2+^ transients and in the contractility in the heart has been extensively evaluated [7, 21, 28, 43, 48, 49, 59, 77, 87, 89, 107, 117]. However, previous reports and the current results shown in Figs. 3 and 4 do not clearly demonstrate the effect of NO transients on Ca^2+^ transients during ECC. To address this unsolved issue, a new protocol was established in which cardiomyocytes received 10 consecutive electrical field stimulations at 2.0 Hz. Once we observed that the NO transient was produced at low frequency, we tested higher frequencies to observe any differences. Among all the frequencies used herein, we did not observe any differences in the production of NO transients. To avoid data analyses of large Ca^2+^ transients due to inactivation and thus an increased sarcoplasmic reticulum Ca^2+^ content [8, 15, 20], only the last six were analyzed. Here, we focus the analyses on the AUC parameter as it summarizes the total effect of NO production on Ca^2+^ transients. During the experiments exemplified in Fig. 5, the AUCs of the Ca^2+^ transients were reduced after a few stimulations but remained constant in the presence of L-NAME in both genotypes, with SMTC showing an effect in WT cardiomyocytes and 1400 W and L-NIO showing effects in dystrophic cardiomyocytes. Furthermore, no significant difference in the intracellular diastolic [Ca^2+^] was observed between WT and mdx cardiomyocytes, suggesting that the negative modulatory effect of the NO transient is limited to the Ca^2+^ transient. Therefore, our results suggested that the isoform-dependent NO transient exerts a negative modulatory effect on the next Ca^2+^ transient according to the pathophysiological condition of the isolated cardiomyocyte. This result supports numerous data obtained by different protocols suggesting that pharmacological nNOS inhibition or nNOS gene disruption significantly increases the Ca^2+^ transient or current amplitude, reducing relaxation and thus increasing contractility [28, 38, 43, 87, 89, 91, 113, 116]. Contrary results have been reported, where knockout nNOS cardiomyocytes showed no increases in Ca^2+^ transients or sarcomere shortening [48, 49]. This discrepancy among data might be due to the ages of the mice (2–3 months old vs. 12 months old) and to the fact that knockout of nNO leads to cardiac hypertrophy, a pathological condition causing an opposite response [6]. Alternatively, compensatory activity from other NOS isoforms is possible, as seen in the heart, i.e., under beta-adrenergic stimulation, wherein eNOS-derived NO increases Ca^2+^ release and reduces Ca^2+^ uptake in sarcoplasmic reticulum vesicles from normal cardiomyocytes [6, 92, 109] and nNOS expression is enhanced in eNOS knockout mice [95].

Previous reports show that NO-dependent Ca^2+^ transient reduction is mediated by two alternative signaling pathways, the NO–cGMP–PKG signaling cascade [28, 69] and SNO [39, 92]. It has been described that both nNOS and eNOs activate sCG-PKG signaling pathway, but due to their microdomain localization and different signaling pathways involved, the nNOS and e-NOS activation does not produce the same effect [4, 114, 115]. Interestingly, under certain circumstances, eNOS and iNOS also share the capability to produce RNS, which results in *S*-nitrosylations [4, 114, 115]. Therefore, it is possible to evaluate the effect of each NOS-derived NO applying directed stimulations. Using specific pharmacological tools for each critical NO signaling point, we further tested which NO pathway was activated by the NO transient to reduce the Ca^2+^ transient in each of the two genotypes. It is important to note that the use of activators of the sGC-PKG signaling pathway does not fully recapitulate the effect of the transient NO production in the cardiomyocyte, but it is one strategy along with others used here to study the role of NO on Ca^2+^ transient. As with L-NAME and SMTC, ODQ significantly increased the Ca^2+^ transient AUC parameter in normal cardiomyocytes, suggesting that nNOS-derived NO was responsible for the negative modulatory effect on Ca^2+^ transients. This result supports and extends previous results showing that NO-activated sGC is responsible for the Ca^2+^ transient reduction and twitch amplitude observed in normal cardiomyocytes [28, 54, 87]. In contrast to what was observed in normal cardiomyocytes, the activation of sGC in dystrophic cardiomyocytes did not produce any differences in comparison to the control solution, suggesting that iNOS- and eNOS-derived NO have alternative signaling pathways. To test this hypothesis, we evaluated one point down in the signaling cascade using KT5823 and 8pCPT as PKG blockers and activators, respectively. We observed an increase in the Ca^2+^ transient AUC parameter in WT cardiomyocytes in the presence of KT5823 and in combination with L-NAME, supporting our data obtained with ODQ and SMTC. These results support a previous report showing that nNOS is the predominant cardiac isoform that controls Ca^2+^ homeostasis, myocyte contraction, and relaxation and signaling pathways, including nitroso-redox balance [28, 54, 87, 89, 114], suggesting that nNOS-derived NO transients act via the NO-sGC-PKG signaling pathway. In mdx cardiomyocytes, activation of PKG with 8pCPT did not increase the Ca^2+^ transient, only in the presence of L-NAME, supporting previous data that iNOS-derived NO transients act via different signaling pathways in dystrophic cardiomyocytes.

The alternative NO signaling pathway was tested using two different SNO blockers, a specific blocker, NEM, and a nonspecific blocker, AA. In contrast to that observed in WT cardiomyocytes, in which neither NEM nor AA reduced the Ca^2+^ transients, these agents did reduce the Ca^2+^ transients in mdx cardiomyocytes. Thus, this result not only further supports and extends the suggested data for the NO-sCG-PKG signaling pathway in WT cardiomyocytes but also proposes an alternative modulatory effect of NO on Ca^2+^ transients in dystrophic cardiomyocytes through iNOS- and e-NOS-dependent SNO.

ROS is a relevant signaling pathway that mediates several responses in normal and dystrophic cardiomyocytes [96]. Interestingly, ROS can be produced by NOS under low levels of l-arginine (< 100 μM) and/or tetrahydrobiopterin [82], generating two major oxidative stress signals: superoxide, which then is converted into peroxynitrite upon reacting with NO [80, 82, 107]. It is established that ROS induces NO production by activating the three isoforms [24, 47, 84]. For instances, brief exposure of HL-1 cardiomyocytes to H_2_O_2_ induces phosphorylation of nNOS at serine 1412, which results in an increased NO production [47]. In neonatal isolated cardiomyocytes, H_2_O_2_-mediated ROS increases iNOS production and NO production [24]. Sartoretto et al. has demonstrated that H_2_O_2_-stimulated eNOS activation depends on phosphorylation of both the AMP-activated protein kinase and kinase Akt, and leads NO production [84]. We tested the effect of exogenous ROS on Ca^2+^ transients incubating H_2_O_2_ in WT cardiomyocytes and using two different ROS scavengers, AA and NAC. In WT cardiomyocytes, H_2_O_2_ did not induce a change in the baseline nor in the NO transient signal, as neither AA nor NAC altered the reduction of the Ca^2+^ transient, as observed with the control solution. Since no NO-dependent ROS production was observed as described by others that used a larger (minutes) time scale [47, 84, 85], these results supports our previous results suggesting that the NO transient signal was triggered after the electrical stimulation. The superoxide also reacts with NO producing peroxynitrite (ONOO^−^) a reactive nitrogen species (RNS), which nitrosylates thiol groups of cysteine promoting significant changes in the activity of the cardiac l-type Ca channel and ryanodine receptor type 2 [65, 70]. The results in mdx cardiomyocytes were similar to those obtained with NEM (RNS scavenger), supporting that the RNS was responsible for the Ca^2+^ transient reduction. These results agree with previous reports showing that ROS are not generated in mdx cardiomyocytes unless they are exposed to osmotic stress, stretch activation, ischemia/reperfusion or mechanical stress [2, 46, 78, 99]. The results regarding basal ROS levels between WT and mdx cardiomyocytes are contradictory, with some studies reporting no difference in ROS content [2, 78] and others reporting a 4.6-fold increase in mdx compared to WT cardiomyocytes [52], despite that the cardiac mitochondrial content is not altered in the mdx mouse heart [52]. The differences might be related to the ages of the mice (22 weeks old in the Kuno et al. study [52], 12 weeks old in the Ascah et al. study [2] and no age reported by Prosser et al. [78]) and/ or the background/ origin of the mdx mice (Japan, Canada or USA). Although our results suggest an effect of RNS on the dystrophic Ca^2+^ transient, some questions remain regarding the sources, amounts and effects of ROS in mdx cardiomyocytes.

In conclusion, we report that NO is transiently produced upon electrical stimulation after the Ca^2+^ transient in isolated murine cardiomyocytes. The evidence presented herein suggests that NO transients are isoform-dependent according to the pathophysiological condition; that is, in WT-isolated cardiomyocytes, the NO transient is derived from nNOS, while in mdx mice, the NO transient is iNOS- and eNOS-dependent as a mechanism compensating for the reduced expression and activity of nNOS. The baseline NO production appears to be iNOS-dependent in both WT and mdx cardiomyocytes, in which nNOS and eNOS contribute, respectively. The major significance of the NO transient observed in isolated cardiomyocytes is the reduction of the subsequent Ca^2+^ transient, leading to a reduced systolic Ca^2+^ transient and thus suggesting how NO modulates contractility.

## Supplementary Information

Below is the link to the electronic supplementary material.

Supplementary file1 All six line-scans from isolated cardiomyocytes. Representative traces obtained from WT cardiomyocytes loaded with NO-ON (10 µM for 2 h) and Rhod-2 (10 µM for 45 min). After 2 s, electrical stimulation (20 V, 10 ms) was provided to simultaneously record the NO fluorescence signal (a) and the Ca^2+^ fluorescence signal (b). The signals were filtered by a simple moving average filter (n=100), and the lines from the same scan are the same color. All six traces are color-coded and identified on the right of the traces. The time scale in seconds is shown at the ordinate, and the fluorescence intensity in arbitrary units is shown at the abscissa. For detailed images and averaged line scans of the same cardiomyocyte, see Fig. 1 (PDF 603 KB)

Supplementary file2 NO transients without Rhod-2 Ca^2+^ fluorescent dye. Representative of three different cardiomyocytes incubated with only NO-ON but excited with both lasers (488 nm for the NO-ON fluorescent dye and 543 nm for the Rhod-2 fluorescent dye) and recorded with both photomultiplier channels (green: 497-537 nm; red: 551-701 nm). Six line-scans of each cell were taken, averaged and filtered by a simple moving average filter (n=100). The green traces show the NO fluorescence signal, and the red traces show the signal without the Rhod-2 fluorescent dye in isolated cardiomyocytes, where the Ca2+ fluorescence signal was normally recorded. Note the difference in the intensities on the red channel in the absence of Rhod-2 versus the presence of Rhod-2 shown in Fig. S2. The time scale in seconds is shown at the ordinate, and the fluorescence intensity in arbitrary units is shown at the abscissa. For experiments with both NO-ON and Rhod-2 dyes, see Figs. 1 and S2 (PDF 472 KB)

Supplementary file3 Complete immunoblot analysis of dystrophin and NOS in isolated cardiomyocytes. Proteins were extracted from cardiomyocytes isolated from five hearts of each genotype. Seventy-five micrograms of protein per lane was used to perform immunoblot analysis of dystrophin (approx. 427 kDa) on a 4-12% gel (a), nNOS (approx. 161 kDa) on a 10% gel (b), iNOS (approx. 133 kDa) on a 10% gel (c), eNOS (approx. 130 kDa) on a 10% gel (d) and GAPDH (approx. 36 kDa) on a 10% gel (e). Nonparametric statistical analyses were performed on the normalized intensity signal, and the raw intensity of GAPDH was used as a loading control (e). **p< 0.01. ns, ns, no significant difference (PDF 1749 KB)

Supplementary file4 Effect of NO on consecutive Ca^2+^ transients. Consecutive Ca^2+^ transients were recorded from cardiomyocytes loaded with Fluo4-AM (5 µM for 30 min) and electrically stimulated (20 V, 10 ms, 2.0 Hz). WT (a) and mdx (b) cardiomyocytes in the presence of different pharmacological agents: control (black and black dotted), 5 µM L-NAME (red and red dotted), 100 nM SMTC (green and green dotted), 1 µM 1400W (blue and blue dotted) and 1 µM L-NIO (orange and orange dotted). Calibration of the Ca^2+^ fluorescence signal was performed on independent isolated cardiomyocytes from the same mice as described in the methods. The time scale in seconds is shown at the ordinate, and the calibrated Ca^2+^ fluorescence intensity in nM is shown at the abscissa. For AUC analysis, see Fig. 3 (PDF 149 KB)

Supplementary file5 H_2_O_2_-derived NO production. Averaged traces from control-treated cardiomyocytes (black; n=7 isolated from four different WT mouse hearts) and 150 µM H_2_O_2_-treated cardiomyocytes (blue; n=11 isolated from 4 different WT mouse hearts). The samples were incubated with H_2_O_2_ for no longer than 5 min. No significant difference was observed between the NO signals recorded from the control and H_2_O_2_-treated cells. (PDF 695 KB)

Supplementary file6 Effect of ROS on consecutive Ca^2+^ transients. Consecutive Ca^2+^ transients were recorded from cardiomyocytes loaded with Fluo4-AM (5 µM for 30 min) and electrically stimulated (20 V, 10 ms, 0.3 and 2.0 Hz). WT (a) and mdx (b) cardiomyocytes in the presence of different pharmacological agents: control (black and black dotted), 10 mM NAC (red and red dotted) and NAC + 5 mM L-NAME. Samples were incubated with the ROS scavenger NAC (10 mM) alone or together with L-NAME for 10 min prior to and during electrical stimulation. Calibration of the Ca2+ fluorescence signal was performed on independent isolated cardiomyocytes from the same mice as described in the methods. The numbers of cardiomyocytes analyzed per treatment obtained from five mice per genotype are shown next to the legend. The symbols *, ** and *** represent p< 0.05, p< 0.01 and p< 0.001, respectively, of the single treatment in comparison to the control. The symbol # represents p< 0.05 of the combined treatment in comparison to the control (PDF 504 KB)

Supplementary file7 (PDF 313 KB)

Supplementary file8 (DOCX 47 KB)

Supplementary file9 (PDF 182 KB)

## Data Availability

The extra-material is available in the supplemental material of the journal.

## Code availability

The code is available upon request.
