# Supplementary figures and images for: Cardiomyocyte depolarization triggers NOS-dependent NO transient after calcium release, reducing the subsequent calcium transient
Source: Basic Res Cardiol. 2021 Mar 17;116(1):18. doi: 10.1007/s00395-021-00860-0 (PMC7966140; doi:10.1007/s00395-021-00860-0)

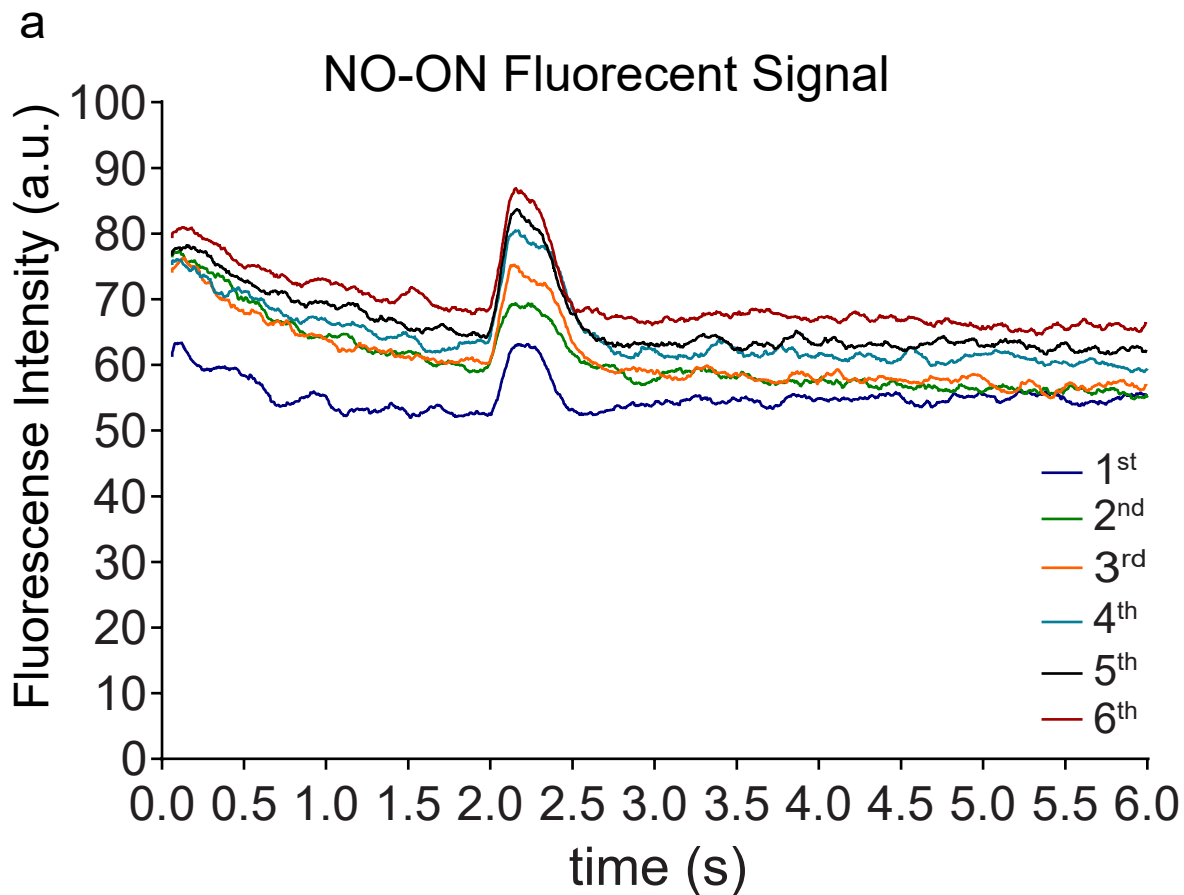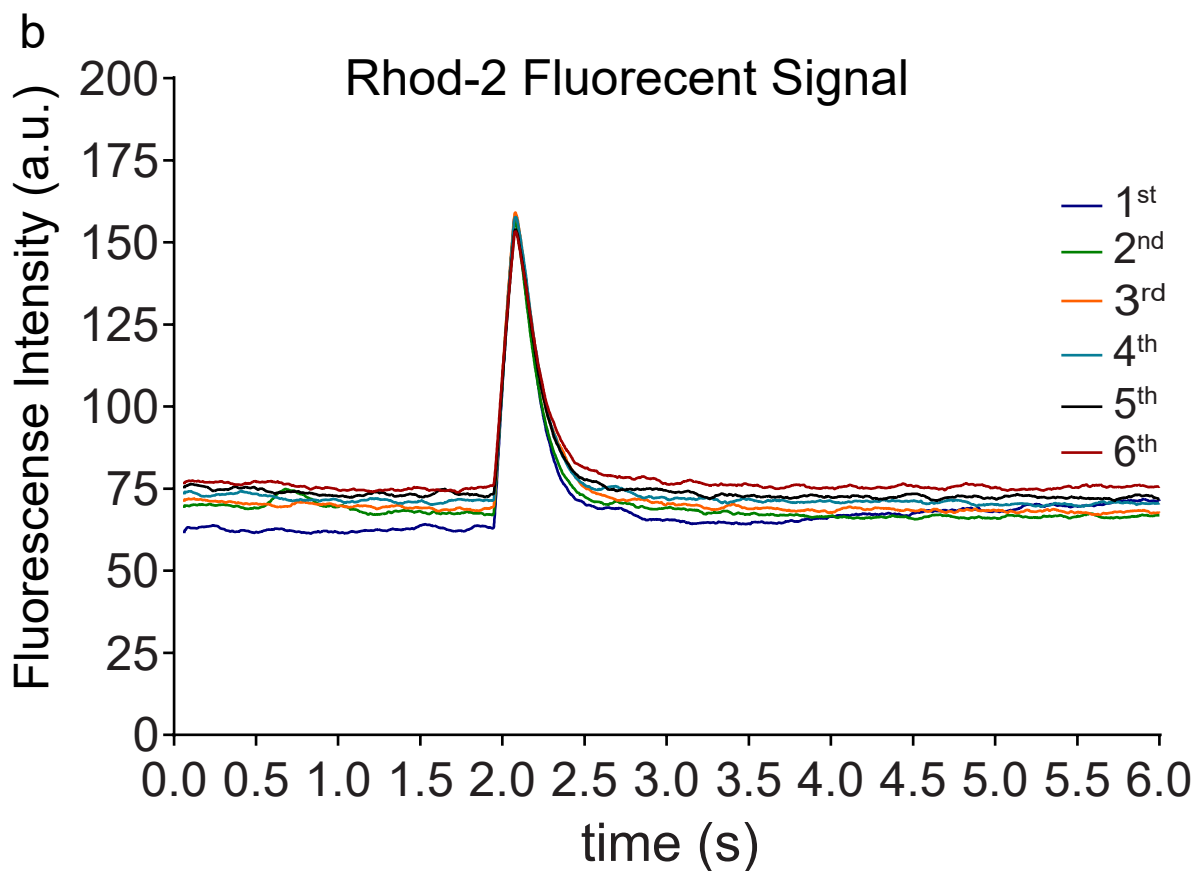

Supplement: Supplementary file 1 — Supplementary file1 All six line-scans from isolated cardiomyocytes. Representative traces obtained from WT cardiomyocytes loaded with NO-ON (10 µM for 2 h) and Rhod-2 (10 µM for 45 min). After 2 s, electrical stimulation (20 V, 10 ms) was provided to simultaneously record the NO fluorescence signal (a) and the Ca2+ fluorescence signal (b). The signals were filtered by a simple moving average filter (n=100), and the lines from the same scan are the same color. All six traces are color-coded and identified on the right of the traces. The time scale in seconds is shown at the ordinate, and the fluorescence intensity in arbitrary units is shown at the abscissa. For detailed images and averaged line scans of the same cardiomyocyte, see Fig. 1 (PDF 603 KB) [file 395_2021_860_MOESM1_ESM.pdf]

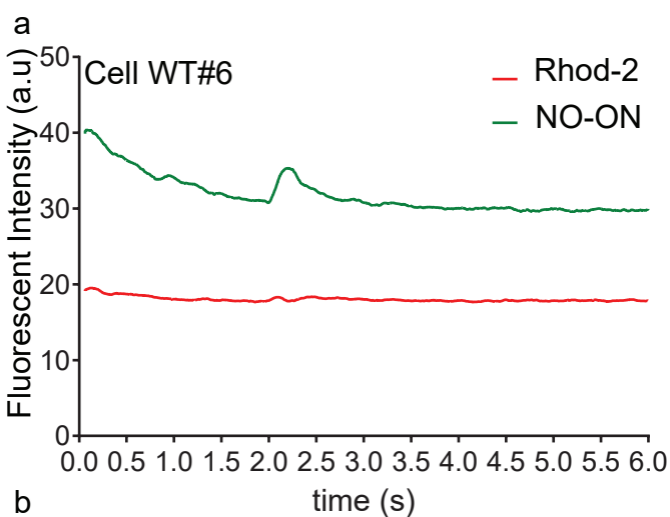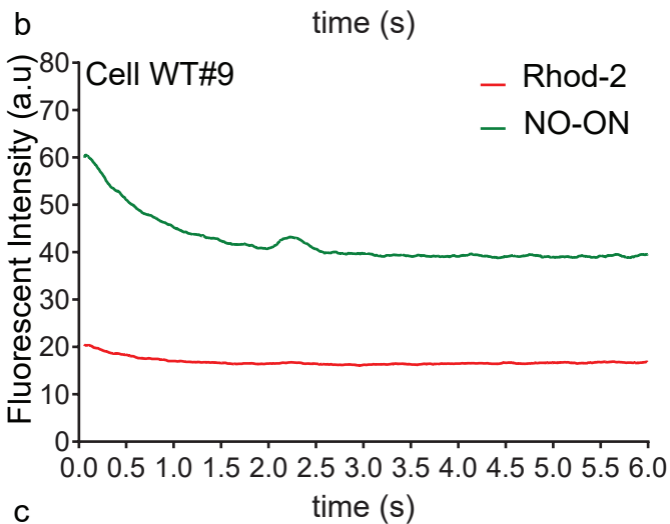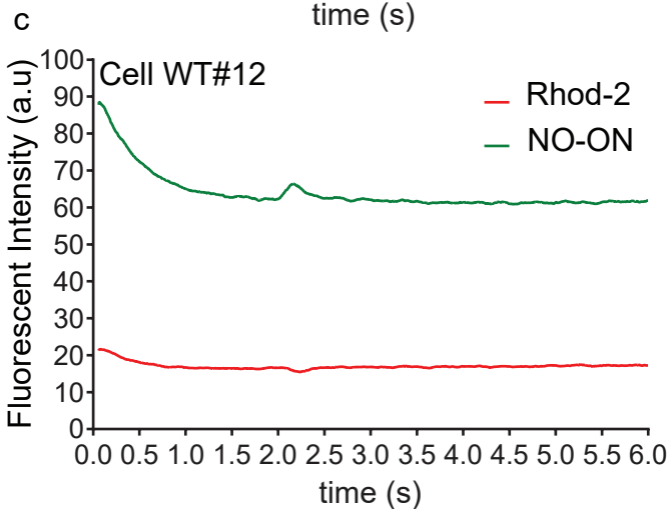

Supplement: Supplementary file 2 — Supplementary file2 NO transients without Rhod-2 Ca2+ fluorescent dye. Representative of three different cardiomyocytes incubated with only NO-ON but excited with both lasers (488 nm for the NO-ON fluorescent dye and 543 nm for the Rhod-2 fluorescent dye) and recorded with both photomultiplier channels (green: 497-537 nm; red: 551-701 nm). Six line-scans of each cell were taken, averaged and filtered by a simple moving average filter (n=100). The green traces show the NO fluorescence signal, and the red traces show the signal without the Rhod-2 fluorescent dye in isolated cardiomyocytes, where the Ca2+ fluorescence signal was normally recorded. Note the difference in the intensities on the red channel in the absence of Rhod-2 versus the presence of Rhod-2 shown in Fig. S2. The time scale in seconds is shown at the ordinate, and the fluorescence intensity in arbitrary units is shown at the abscissa. For experiments with both NO-ON and Rhod-2 dyes, see Figs. 1 and S2 (PDF 472 KB) [file 395_2021_860_MOESM2_ESM.pdf]

A

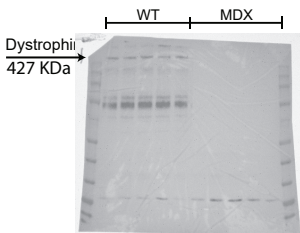

B

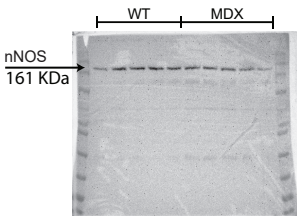

C

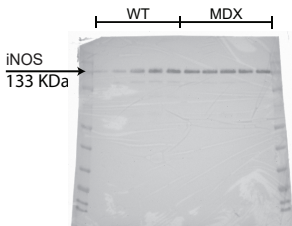

D

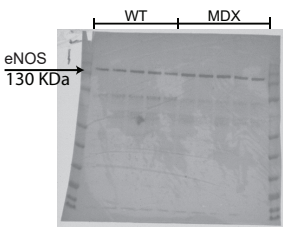

E

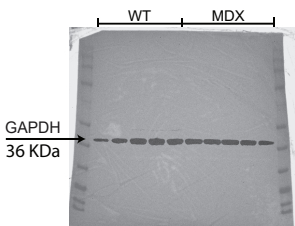

Supplement: Supplementary file 3 — Supplementary file3 Complete immunoblot analysis of dystrophin and NOS in isolated cardiomyocytes. Proteins were extracted from cardiomyocytes isolated from five hearts of each genotype. Seventy-five micrograms of protein per lane was used to perform immunoblot analysis of dystrophin (approx. 427 kDa) on a 4-12% gel (a), nNOS (approx. 161 kDa) on a 10% gel (b), iNOS (approx. 133 kDa) on a 10% gel (c), eNOS (approx. 130 kDa) on a 10% gel (d) and GAPDH (approx. 36 kDa) on a 10% gel (e). Nonparametric statistical analyses were performed on the normalized intensity signal, and the raw intensity of GAPDH was used as a loading control (e). **p< 0.01. ns, ns, no significant difference (PDF 1749 KB) [file 395_2021_860_MOESM3_ESM.pdf]

# NO Signal Traces

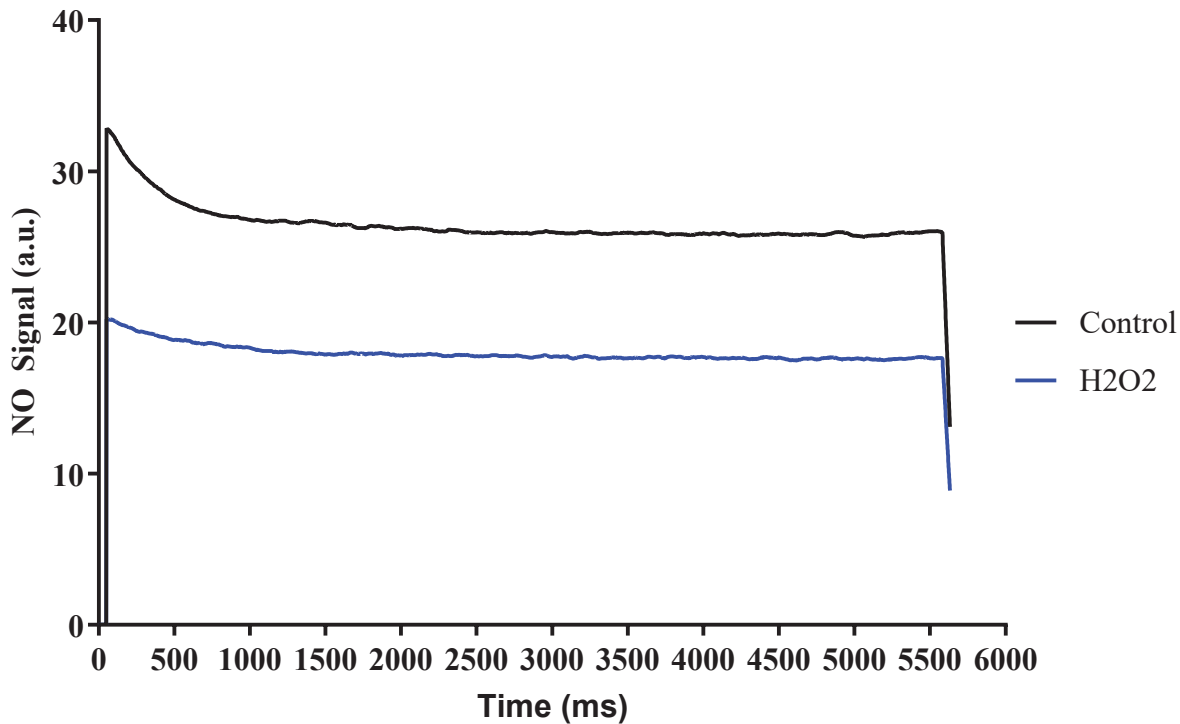

Supplement: Supplementary file 4 — Supplementary file4 Effect of NO on consecutive Ca2+ transients. Consecutive Ca2+ transients were recorded from cardiomyocytes loaded with Fluo4-AM (5 µM for 30 min) and electrically stimulated (20 V, 10 ms, 2.0 Hz). WT (a) and mdx (b) cardiomyocytes in the presence of different pharmacological agents: control (black and black dotted), 5 µM L-NAME (red and red dotted), 100 nM SMTC (green and green dotted), 1 µM 1400W (blue and blue dotted) and 1 µM L-NIO (orange and orange dotted). Calibration of the Ca2+ fluorescence signal was performed on independent isolated cardiomyocytes from the same mice as described in the methods. The time scale in seconds is shown at the ordinate, and the calibrated Ca2+ fluorescence intensity in nM is shown at the abscissa. For AUC analysis, see Fig. 3 (PDF 149 KB) [file 395_2021_860_MOESM4_ESM.pdf]

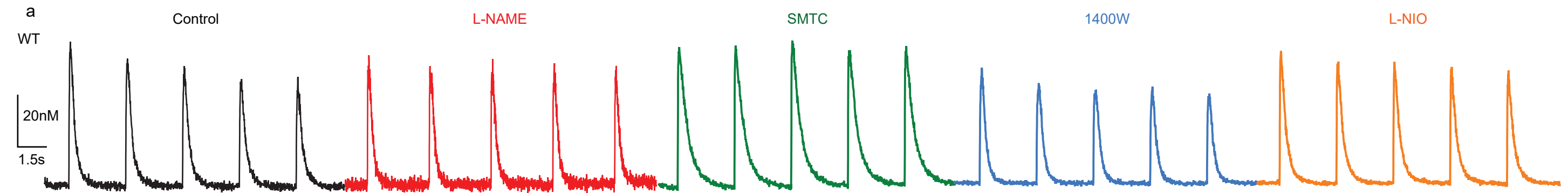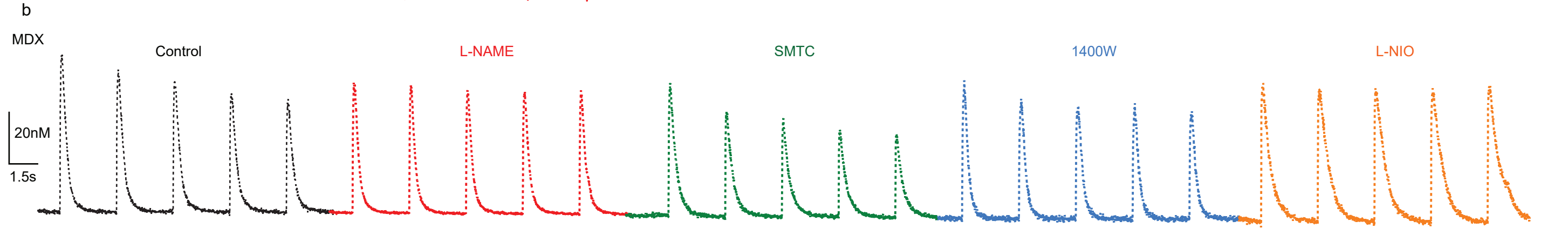

Supplement: Supplementary file 5 — Supplementary file5 H2O2-derived NO production. Averaged traces from control-treated cardiomyocytes (black; n=7 isolated from four different WT mouse hearts) and 150 µM H2O2-treated cardiomyocytes (blue; n=11 isolated from 4 different WT mouse hearts). The samples were incubated with H2O2 for no longer than 5 min. No significant difference was observed between the NO signals recorded from the control and H2O2-treated cells. (PDF 695 KB) [file 395_2021_860_MOESM5_ESM.pdf]

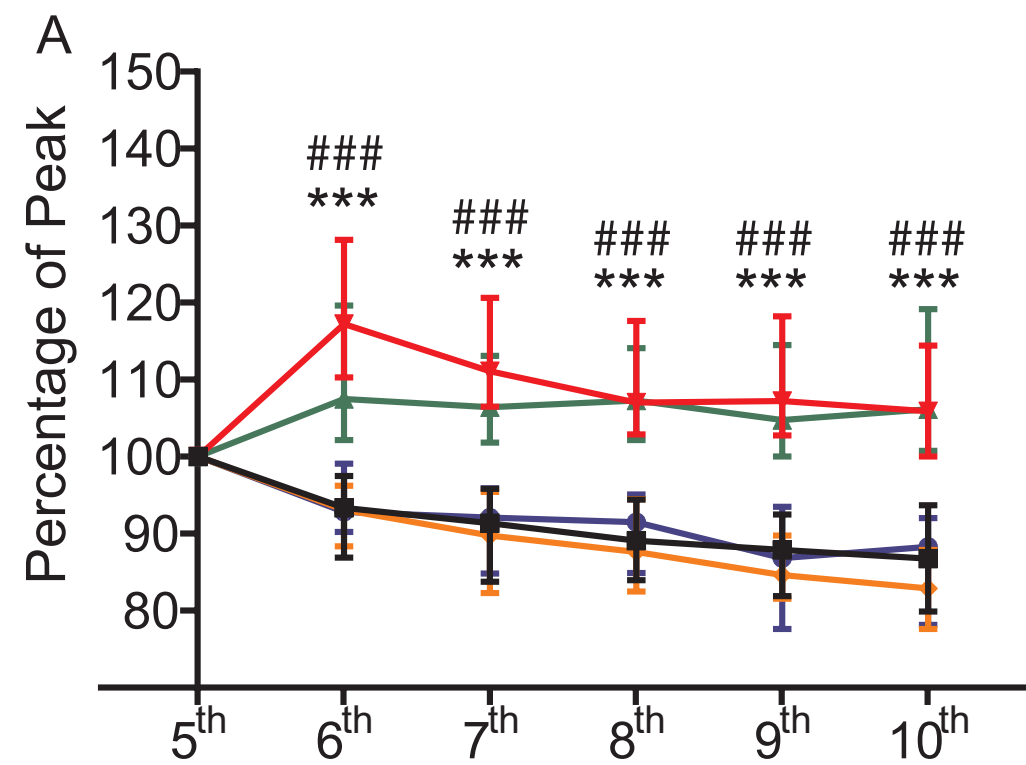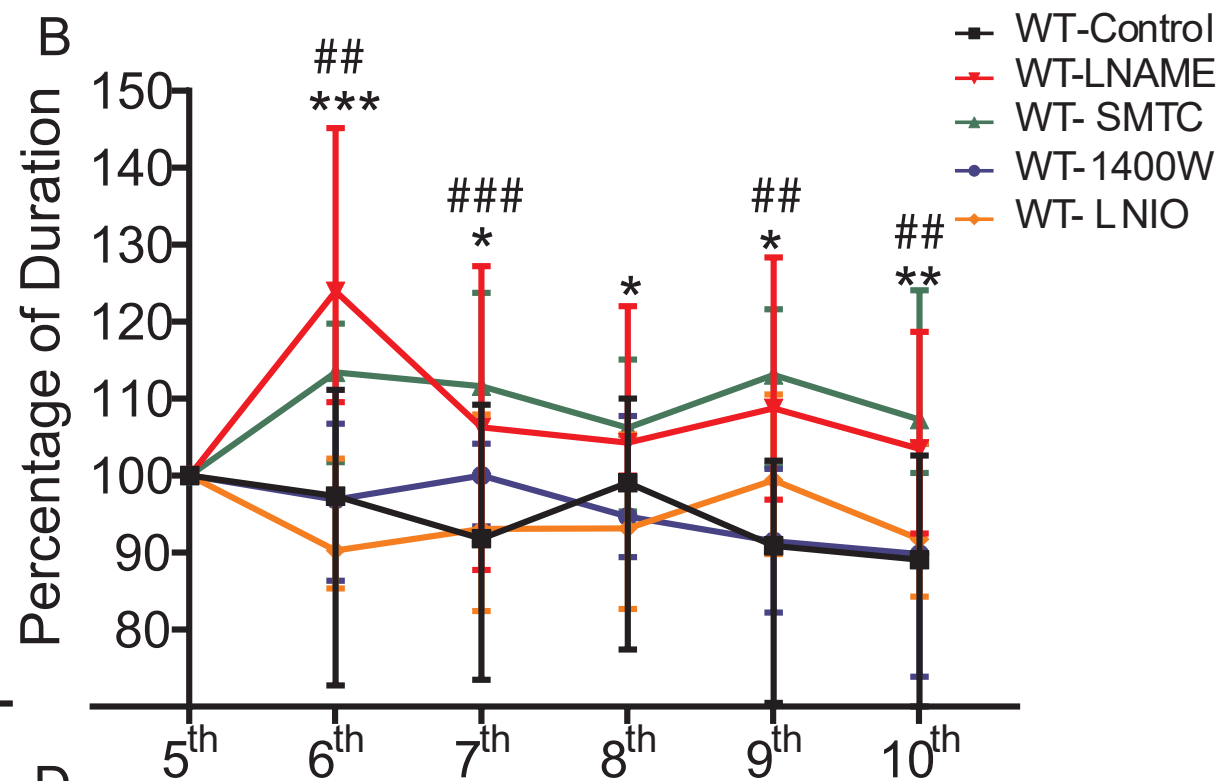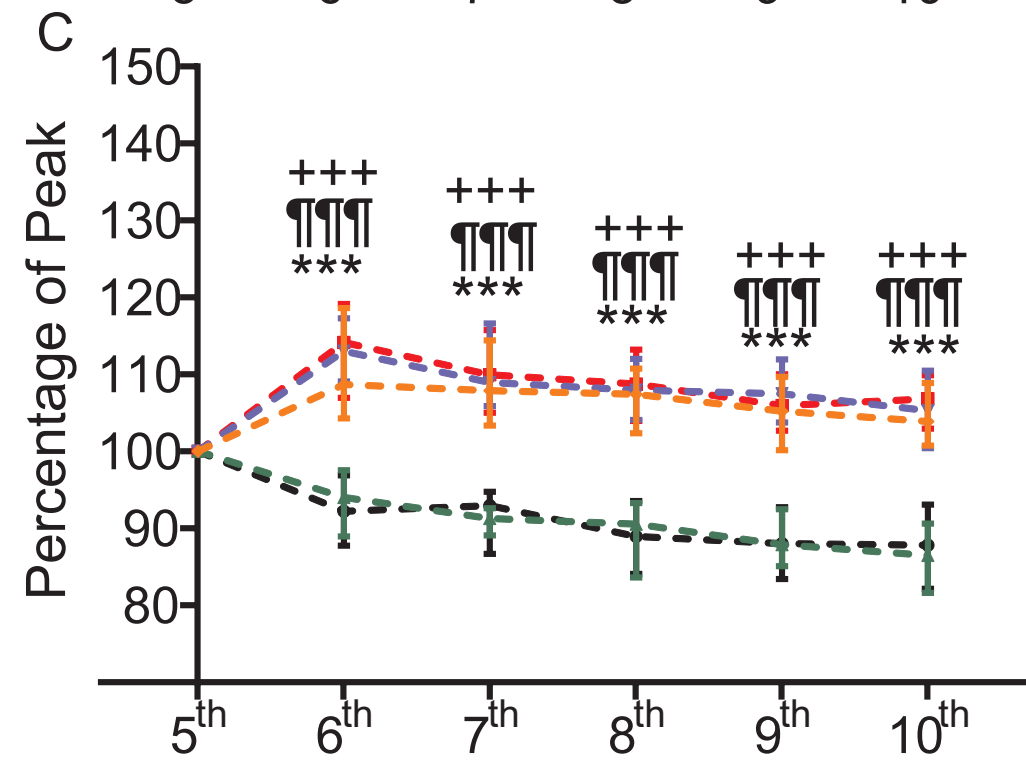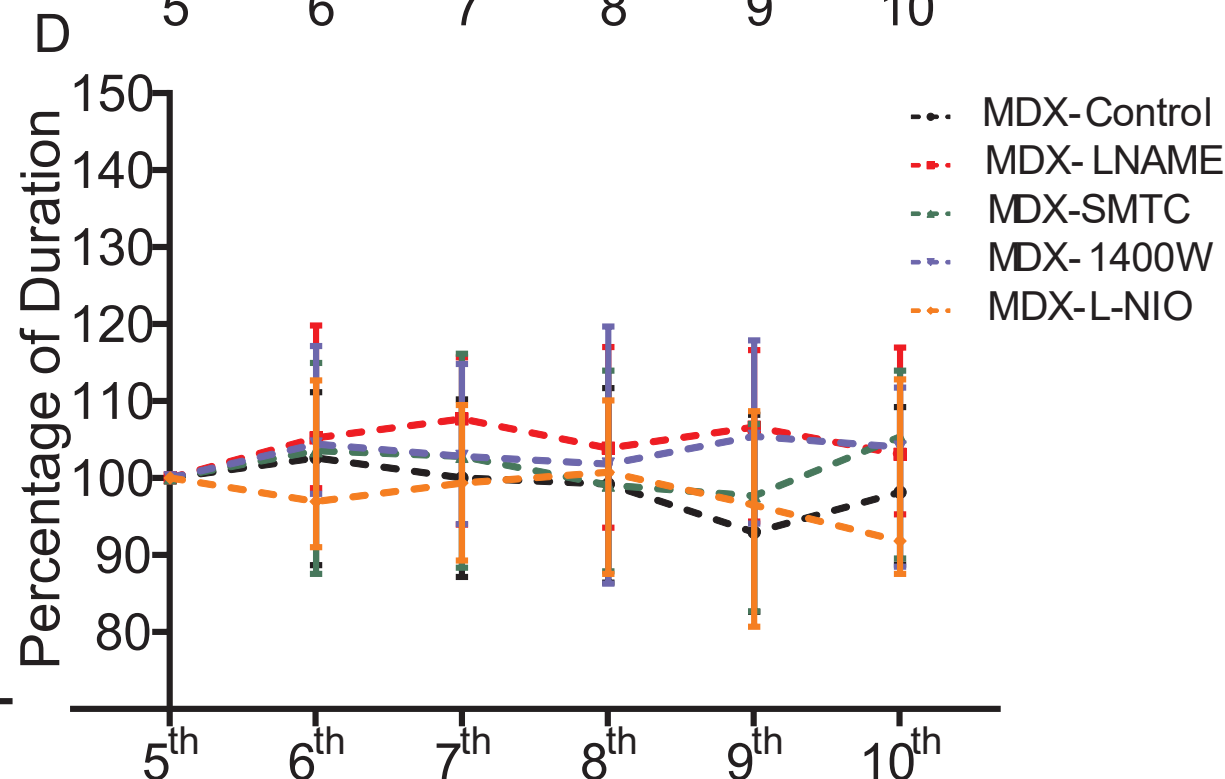

Supplement: Supplementary file 7 — Supplementary file7 (PDF 313 KB) [file 395_2021_860_MOESM7_ESM.pdf]
