# Supplementary material for: Cardiomyocyte depolarization triggers NOS-dependent NO transient after calcium release, reducing the subsequent calcium transient
Source: Basic Res Cardiol. 2021 Mar 17;116(1):18. doi: 10.1007/s00395-021-00860-0 (PMC7966140; doi:10.1007/s00395-021-00860-0)
Supplement: Supplementary file 6 — Supplementary file6 Effect of ROS on consecutive Ca2+ transients. Consecutive Ca2+ transients were recorded from cardiomyocytes loaded with Fluo4-AM (5 µM for 30 min) and electrically stimulated (20 V, 10 ms, 0.3 and 2.0 Hz). WT (a) and mdx (b) cardiomyocytes in the presence of different pharmacological agents: control (black and black dotted), 10 mM NAC (red and red dotted) and NAC + 5 mM L-NAME. Samples were incubated with the ROS scavenger NAC (10 mM) alone or together with L-NAME for 10 min prior to and during electrical stimulation. Calibration of the Ca2+ fluorescence signal was performed on independent isolated cardiomyocytes from the same mice as described in the methods. The numbers of cardiomyocytes analyzed per treatment obtained from five mice per genotype are shown next to the legend. The symbols *, ** and *** represent p< 0.05, p< 0.01 and p< 0.001, respectively, of the single treatment in comparison to the control. The symbol # represents p< 0.05 of the combined treatment in comparison to the control (PDF 504 KB) [file 395_2021_860_MOESM6_ESM.pdf]

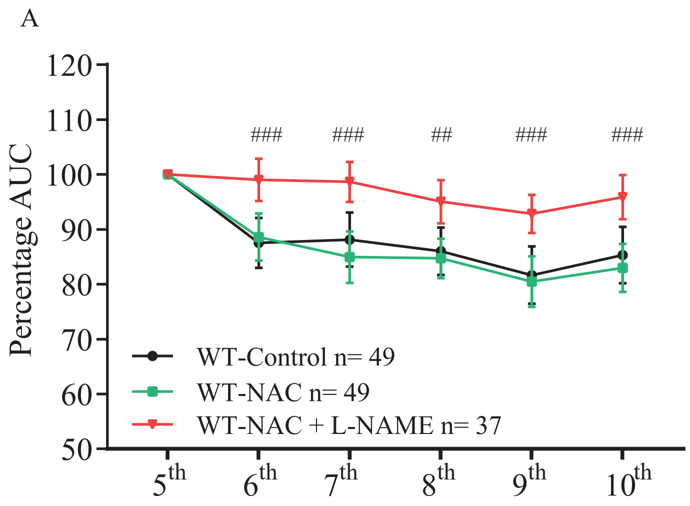

	WT-Control			WT-NAC			WT-NAC + L-NAME		
	Median	Upper Limit	Lower Limmit	Median	Upper Limit	Lower Limmit	Median	Upper Limit	Lower Limmit
5th	100	100	100	100	100	100	100	100	100
6th	91.72	116.82	37.05	93.52	110.85	45.65	96.21	131.93	68.16
7th	91.52	110.72	29.28	89.87	106.16	31.92	98.08	130.63	66.53
8th	88.11	113.16	30.55	88.7	106.4	53.61	92.94	140.41	73.89
9th	83.46	114.97	34.75	80.51	112.67	34.54	91.93	124.29	63.99
10th	87.7	112.41	11.01	85.17	106.66	41.94	92.37	134.84	80.2

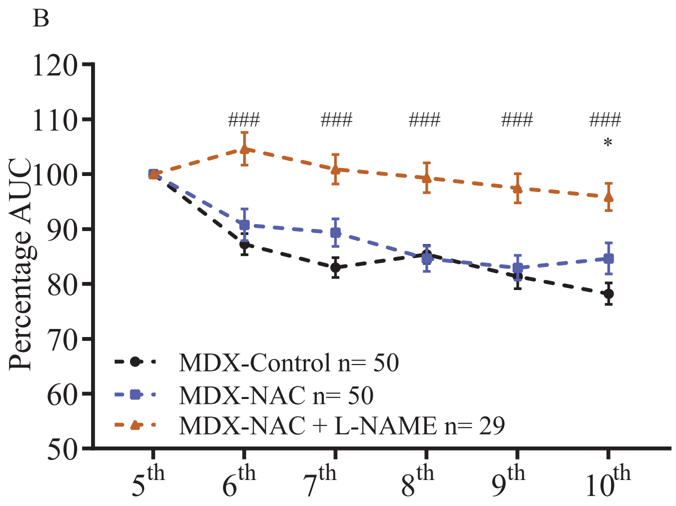

	MDX-Control			MDX-NAC			MDX-NAC + L-NAME		
	Median	Upper Limit	Lower Limit	Median	Upper Limit	Lower Limit	Median	Upper Limit	Lower Limit
5th	100	100	100	100	100	100	100	100	100
6th	87.33	124.1	57.73	92.87	134.26	5.35	100.05	160.39	89.24
7th	85.855	103.52	46.62	88.15	155.79	50.57	97.85	149.63	77.32
8th	86.34	111.19	61.23	84.245	125.86	47.56	96.96	145.03	82.47
9th	83.855	109.3	23.68	80.92	130.58	38.85	96.23	142.91	69.18
10th	79.055	107.35	40.32	83.745	155.79	36.43	98.96	120.96	69.28
