# Supplementary material for: Cardiomyocyte depolarization triggers NOS-dependent NO transient after calcium release, reducing the subsequent calcium transient
Source: Basic Res Cardiol. 2021 Mar 17;116(1):18. doi: 10.1007/s00395-021-00860-0 (PMC7966140; doi:10.1007/s00395-021-00860-0)
Supplement: Supplementary file 8 — Supplementary file8 (DOCX 47 KB) [file 395_2021_860_MOESM8_ESM.docx]

**Supplementary Table S1**. Normalized baseline of NO transient for isolated cardiomyocytes from three WT mice (top) and three MDX mice (bottom). The normalization was calculated the difference between the 6^th^ and the 1^st^ transient divided by the maximum value of the 6^th^ transient from respective treatment.

| **WT** | **Control** | **L-NAME 5mM** | **SMTC 100nM** | **1400W 1µM** | **L-NIO 1µM** | **PTIO 100µM** |
| --- | --- | --- | --- | --- | --- | --- |
| Number of cells | 124 | 50 | 26 | 20 | 25 | 27 |
| Minimum | -0.03 | -0.04 | -0.03 | -0.07 | 0.002 | -0.32 |
| 25% Percentile | 0.03 | -0.008 | 0.01 | -0.02 | 0.06 | -0.21 |
| Median | 0.07 | 0.04 | 0.03 | 0.007 | 0.11 | -0.15 |
| 75% Percentile | 0.14 | 0.10 | 0.07 | 0.06 | 0.22 | -0.03 |
| Maximum | 0.48 | 0.20 | 0.19 | 0.16 | 0.38 | 0.11 |
| F(5,266)= 36.36 |  | p= 0.0113 | p= 0.0111 | p= 0.0011 | p= 0.2073 | p< 0.001 |
| **MDX** | **Control** | **L-NAME 5mM** | **SMTC 100nM** | **1400W 1µM** | **L-NIO 1µM** | **PTIO 100µM** |
| Number of cells | 153 | 27 | 21 | 20 | 24 | 27 |
| Minimum | -0.03 | -0.13 | -0.10 | -0.02 | 0.03 | -0.42 |
| 25% Percentile | 0.01 | -0.048 | -0.007 | -0.010 | 0.07 | -0.14 |
| Median | 0.04 | -0.0004 | 0.02 | -0.002 | 0.13 | -0.053 |
| 75% Percentile | 0.11 | 0.04 | 0.14 | 0.04 | 0.17 | 0.033 |
| Maximum | 0.39 | 0.09 | 0.31 | 0.1 | 0.32 | 0.13 |
| F(5,266)= 18.32 |  | p< 0.001 | p= 6861 | p= 0.0251 | p= 0.0351 | p< 0.001 |

**Supplementary Table S2**. Area under the curve in arbitrary units of NO transient for isolated cardiomyocytes from three WT mice (top) and three MDX mice (bottom). The values are multiplied by 100 to facilitate the reading.

| **WT** | **Control** | **L-NAME 5mM** | **SMTC 100nM** | **1400W 1µM** | **L-NIO 1µM** | **PTIO 100µM** |
| --- | --- | --- | --- | --- | --- | --- |
| Number of cells | 124 | 50 | 26 | 20 | 25 | 27 |
| Minimum | 0 | 0 | 0 | 0 | 0 | 0 |
| 25% Percentile | 0 | 0 | 0 | 0 | 0.07 | 0 |
| Median | 2.02 | 0.80 | 0.43 | 1.90 | 0.42 | 0 |
| 75% Percentile | 5.42 | 2.63 | 3.0 | 8.14 | 3.19 | 0.63 |
| Maximum | 14.91 | 9.52 | 5.33 | 10.89 | 5.80 | 4.50 |
| F(5,266)= 5.119 |  | p= 0.00274 | p= 0.0207 | p= 0.9942 | p= 0.0517 | p= 0.0011 |
| **MDX** | **Control** | **L-NAME 5mM** | **SMTC 100nM** | **1400W 1µM** | **L-NIO 1µM** | **PTIO 100µM** |
| Number of cells | 153 | 27 | 21 | 20 | 24 | 27 |
| Minimum | 0 | 0 | 0 | 0 | 0 | 0 |
| 25% Percentile | 0.001 | 0 | 0.07 | 0 | 0.60 | 0 |
| Median | 2.19 | 0 | 2.07 | 0.05 | 1.47 | 0 |
| 75% Percentile | 5.84 | 0.36 | 4.05 | 2.68 | 2.36 | 1.38 |
| Maximum | 18.31 | 1.68 | 6.54 | 9.16 | 4.02 | 3.63 |
| F(5,266)=8.471 |  | p< 0.0001 | p= 0.1670 | p= 0.0489 | p= 0.0146 | p= 0.0002 |

**Supplementary Table S3**. Area under the curve (AUC) parameter as percentage to the fifth transient from the last 6 out of 10 consecutive Ca^2+^ transients obtained from isolated cardiomyocytes loaded with Fluo4-AM. Detailed data from Figure 5. Values shown in median, upper limit and lower limit. Number of cells “n” recorded from five WT or five MDX mice.

WT: F_INTERACTION(20,952)_=6.46; F_Transient(5,925)_=48.39; F_NOSBlockers(4,185)_=20.06

MDX: F_INTERACTION(20,1165)_=7.12; F_Transient(5,1165)_=31.45; F_NOSBlockers(4,233)_=24.26

| **Treatment** | **Limits** | **5^th^** | **6^th^** | **7^th^** | **8^th^** | **9^th^** | **10^th^** |
| --- | --- | --- | --- | --- | --- | --- | --- |
| WT- Control  n= 49 | Median | 100.00 | 94.69 | 91.11 | 88.92 | 86.13 | 84.56 |
|  | Upper | 100.00 | 110.01 | 102.32 | 106.32 | 99.95 | 100.51 |
|  | Lower | 100.00 | 52.19 | 55.69 | 40.94 | 60.70 | 52.19 |
| WT-  L-NAME  n= 51 | Median | 100.00 | 100.08 | 98.19 | 96.34 | 95.29 | 94.79 |
|  | Upper | 100.00 | 133.60 | 139.02 | 120.97 | 125.25 | 134.89 |
|  | Lower | 100.00 | 81.11 | 73.45 | 71.32 | 44.03 | 65.00 |
|  | P | ns | <0.001 | <0.001 | <0.001 | <0.001 | <0.001 |
| WT –  SMTC  n= 25 | Median | 100.00 | 105.21 | 97.48 | 100.37 | 96.79 | 100.37 |
|  | Upper | 100.00 | 155.57 | 121.89 | 176.13 | 122.35 | 176.13 |
|  | Lower | 100.00 | 89.33 | 82.55 | 86.58 | 69.34 | 76.72 |
|  | P | ns | <0.001 | <0.001 | <0.001 | <0.001 | <0.001 |
| WT - 1400W  n= 38 | Median | 100.00 | 95.22 | 92.85 | 90.26 | 87.33 | 83.56 |
|  | Upper | 100.00 | 113.92 | 110.43 | 109.78 | 103.32 | 108.68 |
|  | Lower | 100.00 | 81.46 | 62.26 | 63.42 | 57.65 | 57.65 |
|  | P | ns | ns | ns | Ns | ns | ns |
| WT –  LNIO  n= 27 | Median | 100.00 | 94.19 | 93.10 | 90.77 | 88.89 | 82.19 |
|  | Upper | 100.00 | 106.55 | 104.50 | 100.28 | 99.11 | 94.52 |
|  | Lower | 100.00 | 62.58 | 63.88 | 42.19 | 62.73 | 42.19 |
|  | P | ns | ns | ns | ns | ns | ns |
| MDX-Control  n= 55 | Median | 100.00 | 94.39 | 93.87 | 89.98 | 90.16 | 89.96 |
|  | Upper | 100.00 | 100.64 | 113.55 | 100.94 | 104.12 | 136.19 |
|  | Lower | 100.00 | 77.47 | 68.14 | 59.33 | 64.55 | 62.22 |
| MDX-  L-NAME  n= 39 | Median | 100.00 | 100.39 | 98.92 | 95.98 | 97.61 | 96.07 |
|  | Upper | 100.00 | 133.60 | 139.02 | 120.97 | 125.25 | 112.64 |
|  | Lower | 100.00 | 84.31 | 73.45 | 71.32 | 44.03 | 65.00 |
|  | P | ns | <0.001 | <0.001 | <0.001 | <0.001 | <0.001 |
| MDX-SMTC  n= 45 | Median | 100.00 | 95.63 | 95.85 | 91.72 | 89.79 | 89.59 |
|  | Upper | 100.00 | 106.28 | 111.40 | 102.93 | 104.05 | 101.34 |
|  | Lower | 100.00 | 57.44 | 57.73 | 63.35 | 58.81 | 63.71 |
|  | P | ns | ns | ns | ns | ns | ns |
| MDX-1400W  n= 54 | Median | 100.00 | 101.99 | 102.22 | 98.85 | 98.61 | 102.70 |
|  | Upper | 100.00 | 133.47 | 140.67 | 123.45 | 136.50 | 128.53 |
|  | Lower | 100.00 | 85.90 | 78.80 | 77.94 | 83.65 | 77.95 |
|  | P | ns | <0.001 | <0.001 | <0.001 | <0.001 | <0.001 |
| MDX-  L-NIO  n= 45 | Median | 100.00 | 100.35 | 98.14 | 97.71 | 97.18 | 101.48 |
|  | Upper | 100.00 | 140.72 | 140.15 | 127.44 | 115.44 | 131.18 |
|  | Lower | 100.00 | 75.40 | 72.20 | 76.53 | 67.20 | 68.13 |
|  | P | ns | <0.001 | <0.001 | <0.001 | <0.001 | <0.001 |

**Supplementary Table S4**. Peak parameter as percentage to the fifth transient from the last 6 out of 10 consecutive Ca^2+^ transients obtained from isolated cardiomyocytes loaded with Fluo4-AM. Detailed data from Figure S7. Values shown in median, upper limit and lower limit. Number of cells “n” recorded from five WT or five MDX mice.

WT: F_INTERACTION(20,952)_=14.11; F_Transient(5,925)_=17.85; F_NOSBlockers(4,185)_=37.53

MDX: F_INTERACTION(20,1165)_=27.26; F_Transient(5,1165)_=50.24; F_NOSBlockers(4,233)_=111.9

| Treatment | Limits | 5^th^ | 6^th^ | 7^th^ | 8^th^ | 9^th^ | 10^th^ |
| --- | --- | --- | --- | --- | --- | --- | --- |
| WT-Control  n=49 | Median | 100 | 93.395 | 91.35 | 89.09 | 87.91 | 86.845 |
|  | Upper | 100 | 106.01 | 103.35 | 108.38 | 102.2 | 100 |
|  | Lower | 100 | 45.16 | 44.65 | 25.51 | 53.57 | 29.7 |
| WT-LNAME  n=51 | Median | 100 | 117.23 | 111.1 | 107.01 | 107.24 | 105.91 |
|  | Upper | 100 | 277.77 | 185.64 | 254.84 | 213.38 | 185.32 |
|  | Lower | 100 | 98.75 | 76.94 | 90.12 | 88.96 | 80.25 |
|  | P | ns |  |  |  |  |  |
| WT-SMTC  n=25 | Median | 100 | 107.49 | 106.46 | 107.32 | 104.79 | 106.17 |
|  | Upper | 100 | 205.85 | 177.63 | 176.65 | 152.58 | 144.33 |
|  | Lower | 100 | 76.27 | 94.77 | 83.8 | 83.74 | 70.16 |
|  | P | ns |  |  |  |  |  |
| WT-1400W  n=38 | Median | 100 | 92.86 | 92.12 | 91.495 | 86.805 | 88.295 |
|  | Upper | 100 | 101.35 | 100 | 102.44 | 104.36 | 115.95 |
|  | Lower | 100 | 79.75 | 74.41 | 64.81 | 54.78 | 53.36 |
|  | P | ns |  |  |  |  |  |
| WT-LNIO  n=27 | Median | 100 | 93.07 | 89.75 | 87.63 | 84.61 | 82.92 |
|  | Upper | 100 | 103.43 | 101.56 | 102.53 | 100 | 100 |
|  | Lower | 100 | 67.33 | 71.8 | 61.13 | 64.56 | 61.9 |
|  | P | ns |  |  |  |  |  |
| MDX Control  n=55 | Median | 100 | 92.23 | 92.95 | 88.99 | 88.05 | 87.86 |
|  | Upper | 100 | 120.46 | 109.36 | 108 | 108.38 | 104.89 |
|  | Lower | 100 | 76.85 | 67.24 | 57.47 | 54.54 | 51.67 |
| MDX-  L-NAME  n= 39 | Median | 100 | 114.185 | 109.965 | 108.775 | 105.935 | 106.85 |
|  | Upper | 100 | 142.84 | 131.66 | 130.51 | 136.3 | 120.45 |
|  | Lower | 100 | 101.28 | 98.6 | 94.37 | 85.54 | 91.29 |
|  | P | ns |  |  |  |  |  |
| MDX- SMTC  n=45 | Median | 100 | 94 | 91.29 | 90.59 | 87.905 | 86.52 |
|  | Upper | 100 | 100.44 | 96.16 | 96.5 | 97.05 | 97.64 |
|  | Lower | 100 | 80.25 | 72.9 | 76.91 | 67.57 | 68.35 |
|  | P | ns |  |  |  |  |  |
| MDX- 1400W  n=54 | Median | 100 | 113.05 | 108.96 | 107.99 | 107.485 | 105.3 |
|  | Upper | 100 | 142.87 | 141.97 | 136.42 | 124.82 | 121.39 |
|  | Lower | 100 | 103.14 | 100.28 | 97.38 | 93.77 | 89.56 |
|  | P | ns |  |  |  |  |  |
| MDX- LNIO  n=45 | Median | 100 | 108.72 | 107.93 | 107.42 | 105.21 | 103.9 |
|  | Upper | 100 | 134.11 | 131.66 | 125.09 | 117.18 | 118.11 |
|  | Lower | 100 | 99.03 | 91.44 | 94.37 | 85.54 | 67.38 |
|  | P | ns |  |  |  |  |  |

**Supplementary Table S4**. Peak parameter as percentage to the fifth transient from the last 6 out of 10 consecutive Ca^2+^ transients obtained from isolated cardiomyocytes loaded with Fluo4-AM. Detailed data from Figure S7. Values shown in median, upper limit and lower limit. Number of cells “n” recorded from five WT or five MDX mice.

WT: F_INTERACTION(20,952)_=2.41; F_Transient(5,925)_=1.71(ns); F_NOSBlockers(4,185)_=7.74

MDX: F_INTERACTION(20,1165)_=0.75(ns); F_Transient(5,1165)_=2.24; F_NOSBlockers(4,233)_=2.26

| Treatment | Limit | 5^th^ | 6^th^ | 7^th^ | 8^th^ | 9^th^ | 10^th^ |
| --- | --- | --- | --- | --- | --- | --- | --- |
| WT- Control  n=49 | Median | 100 | 97.39 | 91.81 | 99.1 | 90.87 | 89.07 |
|  | Upper | 100 | 258.98 | 152.67 | 162.67 | 480.36 | 153.65 |
|  | Lower | 100 | 14.31 | 16.03 | 13.87 | 28.72 | 27.13 |
| WT-LNAME  n=51 | Median | 100 | 123.94 | 106.3 | 104.31 | 108.76 | 103.51 |
|  | Upper | 100 | 172.46 | 145.89 | 156.41 | 185.2 | 176.96 |
|  | Lower | 100 | 67.64 | 68.58 | 60.07 | 47.93 | 60.54 |
|  | P | ns | <0.001 | <0.05 | <0.05 | <0.05 | <0.01 |
| WT – SMTC  n=25 | Median | 100 | 113.44 | 111.61 | 106.23 | 113.11 | 107.33 |
|  | Upper | 100 | 144.12 | 170.07 | 131.7 | 189.05 | 132.86 |
|  | Lower | 100 | 82.12 | 76.19 | 61.35 | 64.71 | 3.09 |
|  | P | ns | <0.01 | <0.001 | ns | <0.01 | <0.01 |
| WT - 1400W  n=38 | Median | 100 | 96.915 | 100 | 94.715 | 91.49 | 89.82 |
|  | Upper | 100 | 143.41 | 159.82 | 137.96 | 137.78 | 141.51 |
|  | Lower | 100 | 34.83 | 63.6 | 41.25 | 49.95 | 34.83 |
|  | P | ns | ns | ns | ns | ns | ns |
| WT – LNIO  n=27 | Median | 100 | 90.33 | 93.11 | 93.16 | 99.43 | 91.78 |
|  | Upper | 100 | 131.75 | 124.56 | 130.48 | 143.11 | 206.01 |
|  | Lower | 100 | 61.48 | 53.81 | 48.13 | 49.34 | 66.06 |
|  | P | ns | ns | ns | ns | ns | ns |
| MDX-Control  n=55 | Median | 100 | 102.62 | 100 | 99.25 | 92.97 | 98.14 |
|  | Upper | 100 | 258.98 | 152.67 | 143 | 170.67 | 168.46 |
|  | Lower | 100 | 55 | 52.13 | 40.37 | 47.91 | 40.37 |
| MDX-LNAME  n=39 | Median | 100 | 105.265 | 107.68 | 103.93 | 106.62 | 103.09 |
|  | Upper | 100 | 268.6 | 184.06 | 172.87 | 161.95 | 166.85 |
|  | Lower | 100 | 66.6 | 76.03 | 70.48 | 71.66 | 73.35 |
|  | P | ns | ns | <0.01 | ns | <0.05 | ns |
| MDX-SMTC  n=45 | Median | 100 | 103.6 | 102.86 | 99.07 | 97.67 | 105.34 |
|  | Upper | 100 | 139.97 | 164.01 | 153 | 156.96 | 144.82 |
|  | Lower | 100 | 68.97 | 65.83 | 64.44 | 66.06 | 57.03 |
|  | P | ns | ns | ns | ns | ns | ns |
| MDX-1400W  n=54 | Median | 100 | 104.41 | 102.82 | 101.85 | 105.445 | 104.01 |
|  | Upper | 100 | 142.35 | 209.47 | 139.75 | 158.88 | 243.42 |
|  | Lower | 100 | 71.06 | 79.58 | 75.3 | 63.02 | 81.2 |
|  | P | ns | ns | ns | ns | ns | ns |
| MDX-  L-NIO  n=45 | Median | 100 | 96.96 | 99.37 | 100.79 | 96.5 | 91.86 |
|  | Upper | 100 | 179.36 | 196.83 | 156.09 | 206.77 | 195.96 |
|  | Lower | 100 | 76.02 | 70.76 | 66.65 | 59.79 | 19.41 |
|  | P | ns | ns | ns | ns | ns | ns |

**Supplementary Table S6**. Pharmacological tools of NO signaling pathway on the percentage of the AUC from the last 6 out of 10 consecutive Ca^2+^ transients normalized to the fifth transient. Detailed data from Figure 6. Values in median, upper limit and lower limits. Number of cells “n” isolated cardiomyocytes loaded with Fluo4-AM recorded from five WT or five MDX mice.

| **Treatment** | **Limits** | **5^th^** | **6^th^** | **7^th^** | **8^th^** | **9^th^** | **10^th^** |
| --- | --- | --- | --- | --- | --- | --- | --- |
| WT-Control n= 30 | Median | 100 | 89.07 | 85.39 | 80.34 | 80.76 | 71.59 |
|  | Upper | 100 | 102.67 | 107.71 | 101.69 | 100.90 | 90.96 |
|  | Lower | 100 | 53.41 | 46.49 | 44.39 | 40.93 | 33.69 |
| WT-ODQ n= 51 | Median | 100 | 91.29 | 86.95 | 86.07 | 81.38 | 80.19 |
|  | Upper | 100 | 137.71 | 134.69 | 138.21 | 127.42 | 138.21 |
|  | Lower | 100 | 54.20 | 62.96 | 60.92 | 57.30 | 23.45 |
|  | P | ns | <0.01 | ns | <0.05 | <0.01 | <0.001 |
| WT-ODQ + L-NAME  n= 28 | Median | 100 | 91.10 | 86.125 | 86.40 | 81.18 | 79.42 |
|  | Upper | 100 | 127.08 | 134.69 | 120.53 | 127.42 | 134.69 |
|  | Lower | 100 | 61.72 | 71.93 | 60.92 | 57.30 | 54.39 |
|  | P | ns | <0.05 | <0.05 | ns | <0.05 | ns |
| WT-Control n=35 | Median | 100 | 88.09 | 93.16 | 88.32 | 86.25 | 87.41 |
|  | Upper | 100 | 110.07 | 108.76 | 107.17 | 108.81 | 104.91 |
|  | Lower | 100 | 29.83 | 37.97 | 25.49 | 13.43 | 29.3 |
| WT-KT5823  n= 50 | Median | 100 | 94.025 | 95.23 | 91.54 | 90.485 | 93.015 |
|  | Upper | 100 | 113.37 | 119.87 | 134.72 | 108.96 | 116.01 |
|  | Lower | 100 | 77.64 | 78.65 | 74.16 | 62.1 | 66.55 |
|  | P | ns | <0.01 | <0.05 | <0.001 | <0.01 | 0.001 |
| WT-KT5823 + L-NAME  n= 31 | Median | 100 | 96.99 | 98.08 | 93.46 | 91.72 | 97.92 |
|  | Upper | 100 | 131.93 | 132.04 | 140.41 | 127.14 | 134.84 |
|  | Lower | 100 | 79.77 | 82.90 | 77.52 | 78.45 | 73.67 |
|  | P | ns | <0.001 | <0.001 | <0.001 | <0.001 | <0.001 |
| WT-Control  n= 30 | Median | 100 | 86.41 | 86.29 | 89.06 | 80.92 | 86.40 |
|  | Upper | 100 | 116.82 | 106.59 | 112.04 | 110.99 | 101.73 |
|  | Lower | 100 | 37.05 | 29.28 | 30.50 | 34.75 | 31.60 |
| WT-NEM  n= 17 | Median | 100 | 90.01 | 83.84 | 79.19 | 78.69 | 82.7 |
|  | Upper | 100 | 110.85 | 106.16 | 106.4 | 110.14 | 103.76 |
|  | Lower | 100 | 45.65 | 31.92 | 59.19 | 34.54 | 41.94 |
|  | P | ns | ns | ns | ns | ns | ns |
| WT-NEM + L-NAME  n= 50 | Median | 100 | 95.37 | 95.38 | 92.85 | 93.21 | 90.68 |
|  | Upper | 100 | 163.82 | 153.65 | 182.18 | 157.16 | 182.90 |
|  | Lower | 100 | 53.13 | 49.64 | 50.59 | 62.37 | 46.97 |
|  | P | ns | <0.01 | <0.001 | <0.05 | <0.001 | <0.01 |
| WT-Control n= 36 | Median | 100 | 92.365 | 91.55 | 87.615 | 83.745 | 87.11 |
|  | Upper | 100 | 103 | 110.72 | 113.16 | 114.97 | 112.41 |
|  | Lower | 100 | 58.88 | 63.61 | 67.48 | 51.70 | 11.01 |
| WT-AA  n= 43 | Median | 100 | 94.21 | 90.91 | 88.70 | 84.57 | 86.27 |
|  | Upper | 100 | 109.59 | 110.76 | 104.92 | 112.67 | 106.66 |
|  | Lower | 100 | 61.64 | 63.84 | 53.61 | 59.26 | 59.40 |
|  | P | ns | ns | ns | ns | ns | ns |
| WT-AA +  L-NAME n=22 | Median | 100 | 104.175 | 100.175 | 95.63 | 98.825 | 98.41 |
|  | Upper | 100 | 130.59 | 147.37 | 110.73 | 114.89 | 113.62 |
|  | Lower | 100 | 86.54 | 90.36 | 76.96 | 82.48 | 73.46 |
|  | P | ns | <0.001 | <0.001 | <0.05 | <0.001 | <0.01 |

**Supplementary Table S7**. Pharmacological tools of NO signaling pathway on the percentage of the AUC from the last 6 out of 10 consecutive Ca^2+^ transients normalized to the fifth transient. Detailed data from Figure 6. Values in median, upper limit and lower limits. Number of cells “n” isolated cardiomyocytes loaded with Fluo4-AM recorded from five WT or five MDX mice.

| **Treatment** | **Limits** | **5^th^** | **6^th^** | **7^th^** | **8^th^** | **9^th^** | **10^th^** |
| --- | --- | --- | --- | --- | --- | --- | --- |
| MDX Control n=31 | Median | 100 | 93.39 | 91.54 | 89.04 | 87.50 | 85.96 |
|  | Upper | 100 | 111.59 | 120.75 | 98.35 | 104.38 | 105.32 |
|  | Lower | 100 | 76.42 | 74.50 | 61.07 | 64.40 | 54.10 |
| MDX-Bay412272 n=28 | Median | 100 | 91.92 | 87.69 | 86.31 | 83.05 | 79.65 |
|  | Upper | 100 | 121.38 | 108.33 | 125.86 | 121.06 | 133.12 |
|  | Lower | 100 | 82.41 | 69.85 | 64.49 | 64.76 | 52.42 |
|  | P | ns | ns | ns | ns | ns | ns |
| MDX-Bay412272+  L-NAME  n= 22 | Median | 100 | 102.87 | 99.64 | 90.745 | 95.13 | 98.98 |
|  | Upper | 100 | 130.57 | 120.84 | 109.73 | 121.99 | 120.49 |
|  | Lower | 100 | 83.69 | 78.36 | 74.12 | 68.46 | 66.49 |
|  | P | ns | <0.001 | <0.001 | <0.05 | <0.01 | <0.001 |
| MDX-Control  n= 54 | Median | 100 | 98.31 | 95.23 | 94.295 | 93.92 | 94.72 |
|  | Upper | 100 | 123.41 | 117.85 | 113.29 | 133.18 | 123.37 |
|  | Lower | 100 | 55.27 | 73.40 | 66.12 | 59.64 | 59.46 |
| MDX-8pCPT  n= 31 | Median | 100 | 98.23 | 94.5 | 93.29 | 92.91 | 89.21 |
|  | Upper | 100 | 119.03 | 117.4 | 119.45 | 114.08 | 107.45 |
|  | Lower | 100 | 87.27 | 73.42 | 73.64 | 73.52 | 73.08 |
|  | P | ns | ns | ns | ns | ns | ns |
| MDX-8pCPT +  L-NAME  n=25 | Median | 100 | 103.77 | 100.41 | 98.83 | 101.27 | 96.96 |
|  | Upper | 100 | 148.29 | 128.62 | 120.40 | 131.03 | 156.94 |
|  | Lower | 100 | 95.71 | 89.13 | 82.77 | 86.22 | 76.93 |
|  | P | ns | <0.001 | <0.001 | <0.05 | <0.001 | <0.01 |
| MDX-Control  n= 37 | Median | 100 | 90.92 | 91.36 | 88.2 | 89.47 | 87.00 |
|  | Upper | 100 | 110.78 | 117.62 | 102.15 | 106.44 | 109.59 |
|  | Lower | 100 | 74.300 | 56.90 | 69.54 | 46.48 | 51.82 |
| MDX-NEM n= 60 | Median | 100 | 96.82 | 95.015 | 94.66 | 95.30 | 95.255 |
|  | Upper | 100 | 121.03 | 107.54 | 112.36 | 121.35 | 115.29 |
|  | Lower | 100 | 76.20 | 66.24 | 61.68 | 54.41 | 47.55 |
|  | P | ns | <0.001 | <0.05 | <0.05 | <0.001 | <0.001 |
| MDX-NEM + L-NAME  n= 60 | Median | 100 | 95.58 | 94.02 | 92.68 | 93.47 | 92.24 |
|  | Upper | 100 | 126.02 | 106.64 | 112.36 | 110.47 | 115.29 |
|  | Lower | 100 | 74.30 | 77.62 | 76.51 | 63.93 | 68.86 |
|  | P | ns | <0.01 | <0.05 | <0.01 | <0.01 | <0.01 |
| MDX-Control  n= 35 | Median | 100 | 84.73 | 83.21 | 85.60 | 82.09 | 76.39 |
|  | Upper | 100 | 124.10 | 102.11 | 111.19 | 109.30 | 107.35 |
|  | Lower | 100 | 57.73 | 46.62 | 43.93 | 23.68 | 40.32 |
| MDX-AA  n= 50 | Median | 100 | 93.19 | 88.60 | 83.76 | 80.57 | 86.87 |
|  | Upper | 100 | 176.31 | 155.79 | 133.12 | 155.58 | 176.31 |
|  | Lower | 100 | 50.35 | 50.57 | 47.56 | 38.85 | 36.43 |
|  | P | ns | ns | <0.05 | ns | ns | <0.001 |
| MDX-AA + L-NAME  n= 25 | Median | 100 | 98.79 | 92.71 | 92.10 | 93.43 | 93.87 |
|  | Upper | 100 | 201.47 | 160.52 | 145.03 | 147.62 | 160.52 |
|  | Lower | 100 | 75.27 | 52.48 | 64.93 | 56.67 | 60.46 |
|  | P | ns | <0.001 | <0.001 | <0.01 | <0.001 | <0.001 |

**Supplementary Table S8. List of reagents.**

| Symbol | Name | Concentration | Action | Company | Cat No. |
| --- | --- | --- | --- | --- | --- |
| L-NAME | Nω-Nitro-L-Arginine methyl-esther hydrochloride | 5mM | Unspecific NOS blocker | Sigma-Aldrich | N5751 |
| SMTC | S-Methyl-L-thiocitrulline acetate salt | 100nM | nNOS blocker | Sigma-Aldrich | M5171 |
| 1400W | N-(3-[Aminomethyl]benzyl) acetamidine | 1µM | iNOS blocker | Sigma-Aldrich | W 4262 |
| L-NIO | L-N5-(1-Iminoethyl) ornithine, Dihydrochloride | 1µM | eNOS blocker | Calbiochem/ Merk-Millipore | 400600 |
| PTIO | Carboxy-PTIO potassium salt | 200µM | NO Scavenger | Sigma-Aldrich | C221 |
| ODQ | 1H-[1,2,4]Oxadiazolo[4,3-a]quinoxalin-1-one | 10 μM | sGC Blocker | Sigma-Aldrich | O3636 |
| Bay412272 | 3-(4-Amino-5-cyclopropylpyrimidin-2-yl)-1-(2-fluorobenzyl)-1H-pyrazolo[3,4-b]pyridine | 10 μM | sGC Activator | Sigma-Aldrich | B8810 |
| KT5823 | 9S,10R,12R)-2,3,9,10,11,12-Hexahydro-10-methoxy-2,9-dimethyl-1-oxo-9,12-epoxy-1Hdiindolo[  1,2,3-fg:3',2',1'-kl]pyrrolo[3,4-i][1,6]benzodiazocine-10-carboxylic acid, methyl ester | 1 μM | PKG Blocker | ABCAM | KT5823 ab120423 |
| 8pCPT | 8-(4-Chlorophenylthio)-2′-O-methyladenosine 3′,5′-cyclic monophosphate monosodium hydrate | 10μM | PKG Activator | Sigma-Aldrich | C5438 |
| NEM | N-Ethylmaleimide | 20μM | SNO Blocker | Sigma-Aldrich | E3876 |
| AA | L-Ascorbic acid | 1 mM | SNO Blocker | Sigma-Aldrich | A1300000 |
| NAC | N-acetylcyteine | 10 mM | ROS blocker | Sigma-Aldrich | A7250 |
