# Supplementary material for: Cardiomyocyte depolarization triggers NOS-dependent NO transient after calcium release, reducing the subsequent calcium transient
Source: Basic Res Cardiol. 2021 Mar 17;116(1):18. doi: 10.1007/s00395-021-00860-0 (PMC7966140; doi:10.1007/s00395-021-00860-0)
Supplement: Supplementary file 9 — Supplementary file9 (PDF 182 KB) [file 395_2021_860_MOESM9_ESM.pdf]

Cardiomyocyte depolarization triggers NOS-dependent NO transient reducing the following calcium transient.

Roland Konietzny[1]#, Carolin Andresen[1,2], Chao Wang[1,3], Rainer HA Fink[1], Matias Mosqueira[1]#*.

1. Cardio-Ventilatory Muscle Physiology Laboratory, Institute of Physiology and Pathophysiology, University Hospital Heidelberg. 69120 Heidelberg, Germany.

2. Medical Biophysics Unit, Institute of Physiology and Pathophysiology, University Hospital Heidelberg. 69120 Heidelberg, Germany.

2. Current address: Heidelberg Institute for Stem Cell Technology and Experimental Medicine (HI-STEM gGmbH), Heidelberg, Germany. Division of Stem Cells and Cancer, German Cancer Research Center (DKFZ), Heidelberg, Germany

3. Current address: Cardiovascular Department, Wuhan No. 1 Hospital, Hubei, China

Supplemental Information Material & Methods

Material and Methods

Mice

Wild type (C57BL/10ScSn) and MDX (C57BL/10ScSn-Dmd^{mdx}/J) age-matched (average age 54 weeks) mice were used for the experiments. All experiments have been approved by the ethics committee of the University of Heidelberg Interfaculty Biomedical Research Facility (T-83/14 and T-21/17) and according to the guidelines of the Regierungspräsidium Karlsruhe of State of Baden-Wuerttemberg.

Cell isolation

Single cardiomyocyte isolation using Langendorff perfusion set up were previously described[85,121] with few modifications. On the day of experiments all solutions were freshly prepared and the pH value were adjusted with 1.0 M NaOH. The animals were heparinized via i.p. 20 minutes before sacrifice via cervical dislocation. The isolated heart was cannulated (G20) in between the atria and suturing the atria onto the cannula to hold the heart. The excess of blood was then removed from the ventricles using Perfusion Solution (in mM: 135 NaCl, 4 KCl, 1 $MgCl_2$, 10 HEPES, 0.33 NaH_2PO_4, 10 Glucose, 20 BDM, 5 Taurine, pH 7.2) and immediately connected to the 37 °C pre warmed Langendorff set up. After 5 min of flow of Perfusion solution, the heart was perfused with Digestion Solution (Collagenase D, Roche, cat no. 11088858001, 0.36mg/g of mouse; Collagenase

B, Roche, cat no. 11088807001, 0.48mg/g of mouse and Protease from *Streptomyces griseus* type XIV, Sigma-Aldrich, cat no. P5147-100MG, 0.06mg/g of mouse; dissolved in 30ml of Perfusion Solution) for 20-30 min. Once the heart was soft and paled, the heart was removed from the Langendorff set up and placed into a 35mm plate with TB-A solution (in mM: 135 NaCl, 4 KCl, 1 $MgCl_2$, 10 HEPES, 0.33 NaH_2PO_4, 5.5 Glucose, 15 BDM, 5mg/ml BSA, pH 7.2) and gently mechanically dissociated with forceps. The single isolated cardiomyocytes were then seeded into 35 mm imaging petri dish (Zell-Kontakt, cat no. 5160-168) pre-coated with 50 µl of ECM gel (Engelbreth-Holm-Swarm murine sarcoma, Sigma-Aldrich, cat. no. E1270-5ML). In three steps of five minutes each, the Ca^{2+} concentration was increased from 0.24, 0.6 to 1.2 mM mixing TB-A and TB-B (in mM: 137 NaCl, 5.4 KCl, 1.8 $CaCl_2$, 1 $MgCl_2$, 10 HEPES, 5.5 Glucose, pH 7.4). The cardiomyocytes were stored in the physiological Ca^{2+} concentration of 1.8 mM in TB-B solution at 37°C and 5% CO_2 in the incubator and were used for experiments on the same day.

Fluorescence dyes and pharmacological agents

The cells were incubated with fluorescence dyes and pharmacological agents at 37°C and 5%CO_2. The cell permanent Ca^{2+} fluorescence dye Rhod-2-AM (Invitrogen, cat no. R1245MP) was dissolved in DMSO and the determined optimal working concentration in TB-B was 10µM with an incubation time of 45 minutes. For intracellular NO-measurements the cardiomyocytes were prepared with the trappable NO dye Cu_2(FL2E) (Nitric Oxide Sensor Intracellular Kit "NO-ON"-FL2E, Strem Chemicals, cat no. 96-0396). As recommend by the manufacturer (www.strem.com) the FL2E powder was dissolved in DMSO for a 1mM stock solution and freshly mixed with 1mM Copper(II)-solution in a 1:1 ratio before incubation started. The determined optimal working concentration in TB-B was 10µM with an incubation time of 2 hours.

The incubation time mentioned for each drug corresponds to the time for the optimal result before the beginning of the experiment and the same concentration of the drug was kept during the entire experiment. For control experiments the cardiomyocytes were incubated with 200µM L-Arginine (L-Arginine monohydrochloride, Sigma-Aldrich, cat no. A5131-10G) for 20 minutes. All drugs mentioned below were further dissolved in control solution containing 200 µM L-Arginine. To block unspecifically all nitric oxide synthases (NOS), the cardiomyocytes were incubated with 5mM L-NAME (Nω-Nitro-L-Arginine methyl-esther hydrochloride, Sigma-Aldrich, cat no. N5751-10G) for 45min. The neuronal nitric oxide synthase (nNOS) was blocked with 100nM

SMTC (S-Methyl-L-thiocitrulline acetate salt, Sigma-Aldrich, cat no. M5171-10MG) with an incubation time of 30 minutes[56,122,123]. The inducible nitric oxide synthase was block with 1µM 1400W (N-(3-[Aminomethyl]benzyl)acetamidine, Sigma-Aldrich, cat no. W4262-10MG) during 30 minutes before the experiment[57,124]. The endothelial nitric oxide synthase was blocked with the incubation for 1 hour with 1µM L-NIO (L-N5-(1-Iminoethyl)ornithine, Dihydrochloride, Calbiochem/ Merk-Millipore, cat no.400600-20mg)[116,125,126]. To scavenge NO, 200µM PTIO (Carboxy-PTIO potassium salt, Sigma-Aldrich, cat no. C221-10MG) was incubated for 1h[127,128].

Detailed description

NO-ON fluorescence dye

Nitric Oxide Sensor (Intracellular) Kit ("NO-ON") (FL2E) (Cell-trappable NO fluorescent probe) from Strem Chemicals, cat no. 96-0396

Includes:

5 x 1ml Dimethylsulfoxide (ACS spectrophotometric grade)

5 x 1ml Copper (II) chloride as a 1.0 mM solution in water

5 x 0.5mg FL2E (powder)

Kit stored at -20°C

FL2E stock solution

FL2E powder	[ ] mM	MW g/mol	Mass µg	DMSO µl
	1	875.66	500	571

In one vial of 0.5mg FL2E powder are diluted in 571µl DMSO and aliquot in 2µl for storing at -20°C. Before incubation: 2µl of FL2E stock solution were freshly mixed with 2µl of 1mM Copper solution (1:1 ratio)

Working solution NO-ON: For the 10µM final concentration, dilute 2µl of the 1:1 mixed FL2E and Copper solution in 200µl TB-B and incubate them at 37°C for 2h

Rhod-2 stock solution

**Rhod-2 AM**	**[ ] mM**	**MW g/mol**	**Mass µg**	**DMSO µl**
	1	1123.96	50	44,8

Rhod-2 AM 50µg are diluted in 44.8µl steril DMSO and aliquot in 2µl for storing at -20°C

Working Solution Rhod-2 AM: For the 10µM final concentration, dilute 2µl of Rhod-2 AM 1mM stock solution in 200µl of TB-B and incubate them at 37°C for 45min

Drugs

L-Arginine (L-Arginin monohydrochloride, Sigma-Aldrich, cat no. A5131-10G) all control solutions (TB-B) contained 200µM L-Arginine L-NAME (Nω-Nitro-L-Arginine methyl-esther hydrochloride, Sigma-Aldrich, cat no. N5751-10G) an unspecific blocker of all nitric oxide synthases (NOS), 5mM working concentration dissolved in control solution (TB-B).
SMTC (S-Methyl-L-thiocitrulline acetate salt, Sigma-Aldrich, cat no. M5171-10MG) a specific blocker of neuronal nitric oxide synthases (nNOS), 100nM working concentration dissolved in control solution (TB-B).
1400W (N-(3-[Aminomethyl]benzyl)acetamidine, Sigma-Aldrich, cat no. W4262-10MG) a specific blocker of inducible nitric oxide synthases (iNOS), 1µM working concentration dissolved in control solution (TB-B).
L-NIO (L-N5-(1-Iminoethyl)ornithine, Dihydrochloride, Calbiochem/ Merk-Millipore, cat no.400600-20MG) a specific blocker of endothelial nitric oxide synthases (eNOS), 1µM working concentration dissolved in control solution (TB-B).
PTIO (Carboxy-PTIO potassium salt, Sigma-Aldrich, cat no. C221-10MG) a scavenger of nitric oxide (NO), 200µM working concentration dissolved in control solution (TB-B).

Stocks: All stocks were stored at -20°C

**Stock (Drug)**	**concentration**	**MW g/mol**	**Mass mg**	**Solution**	**disolved in**
L-Arginin	10 mg/ml	210.66	5	500 µl	TB-B
SMTC	10 mM	265.33	10	3.768 ml	Millipore water
1400W	1 mM	250.2	0.5 mg	2 ml	DMSO
L-NIO	20 mM	246.1	20 mg	4.06 ml	DMSO
PTIO	20 mM	315.39	10 mg	1.58 ml	DMSO
**Stock 2 (Drug)**	**concentration**	**MW g/mol**	**used Stock 1 µl**	**Solution**	**disolved in**
SMTC	100 µM	265.33	10 µl	1ml	Millipore water
L-NIO	1 mM	246.1	3 µl	60 µl	DMSO

Working Solutions:
All working solutions were prepared freshly on the day of experiments
The incubation time corresponds to the time for best results before the beginning of experiments.
The same concentration of drug was kept during the experiments.

**Drug**	**concentration**	**MW g/mol**	**used stock µl**	**TB-B µl**	**Incubation Time**

L-Arginin	200 µM	210.66	42.1 µl	10 ml	20 min
SMTC	100 nM	265.33	5 µl of stock 2	5 ml	30 min
1400W	1 µM	250.2	5 µl	5 ml	30 min
L-NIO	1 µM	246.1	5 µl of stock 2	5 ml	60 min
PTIO	200 µM	265.33	50 µl	5 ml	60 min
**Drug**	**concentration**	**MW g/mol**	**Mass mg**	**TB-B ml**	**Incubation Time**
L-NAME	5 mM	269.69	13.5	10	45 min

**TB-B 50 ml**	**[ ] mM**	**MW g/mol**	**Mass mg**
NaCl	137	58.44	400.31
KCl	5.4	74.56	20.13
CaCl2*2H2O	1.8	147.0146	13.23
MgCl2*6H2O	1	203.30	10.16
HEPES	10	238.31	119.64
Glucose	5.5	180.16	50.00
pH	7.4 at 37°C		

Confocal microscopy and data acquisition (measurement procedure)

A confocal laser scanning microscope (Leica TCS MR 2) with a 63x water immersion objective (PL Apo 63x/1.20 W CORR from Leica) was used. The NO fluorescence dye FL2E was excited by the Argon laser line 488nm and the emission was detected by a photomultiplier in the spectral range of 497-537nm. The Ca^{2+} fluorescence dye Rhod 2 was excited with the He/Ne laser line 543nm and the emission light was detected by a photomultiplier between 551-701nm. All images were saved as 8-bit images and had a pixel size of 0.186µm and a time resolution of 800 lines per second. XYT images contained 512*512 pixels and XT images recorded 5632 lines with 512 pixels per line. The line scan trace was set at the center of the cardiomyocyte and away from the nucleus. Two seconds after the initial of the line scan, the cardiomyocytes were stimulated with a square pulse of about 20V and 10ms duration via two bath platinum electrodes connected to a stimulator (Stimulator, SI Heidelberg). Six line scans were recorded per cell with an interval of 6 seconds between each line scan. The images were averaged and filtered offline by an unweighted moving average (n=100) reducing noise. Traces were normalized to baseline by an exponential decay fit and analyzed with a custom-made code in Fiji and python3.5.2 (spyder, anaconda3) using the physiological parameters: area under the curve (AUC) and delta time start (starting time difference

between Ca^{2+} and NO Transient). Computer code is fully available upon request. For baseline analysis the value of the first baseline were subtracted from the sixth baseline and normalized by dividing by the highest value of the sixth baseline for each group of drugs.

Consecutive Ca^{2+} transients

Cardiomyocytes obtained as described above from different mice were used to record consecutive Ca^{2+} transients in a different set up, as previously described[85]. Cardiomyocytes from each heart were seeded in four 35 mm imaging petri dish and incubated during 30 min with 5 µM Fluo-4-AM (Life Technologies, Carlsbad, CA, USA) dissolved in TB-B. Three 35 mm imaging petri dish were used for recording consecutive Ca^{2+} transients before and after different treatments and one for Ca^{2+} calibration. From each 35 mm imaging petri dish, a minimum of 10 cardiomyocytes were recorded as controls and a minimum of 15 cells were recorded after drug application on each plate. The consecutive Ca^{2+} transients were recorded using an Olympus OSP-3 System, 2nd Generation microscope connected to a Photomultiplier unit (Olympus, Tokyo, Japan) at 20x magnification equipped with a Xenon light source system, filtered to provide an excitation wavelength of 488 nm. A recording area of 7.5 μm^2 and away from the nucleus was selected on the photomultiplier unit's pinhole. Cardiomyocytes were flanked by a pair of platinum electrodes connected to a SD9 Gras Instruments stimulator and single-twitch stimulations at 20 V, during 10 ms at frequency of 0.20 Hz. TB-B were used as control solution and to dissolve other drugs. Only cardiomyocytes having a stable response for the electrical stimulation were selected and the fluorescence intensity of 8-10 transients was recorded. Subsequently, TB-B was removed and 2 ml of the respective drug dissolved in TB-B was added into the 35 mm imaging petri dish. After a period of 10 minutes of incubation, consecutive Ca^{2+} transients were recorded as described above. The fluorescence intensity was sampled at 2KB/s in a PC using LabChart Pro V8 (AD Instruments). The fourth 35 mm petri dish were used to convert Fluo-4 AM fluorescence intensity into intracellular Ca^{2+} concentration ($[Ca^{2+}]_i$), as described previously[68,129]. Briefly, the cells were incubated to a high Ca^{2+}-concentration solution that contained (in mM) 140 LiCl, 5 KCl, 1.2 KH_2PO_4, 1.2 $MgCl_2$, 4 $CaCl_2$, 20 HEPES, 0 EGTA, 0.005 Ionomycin, 0.01 CPA, 5 Caffeine and 1 Oubain and the resulting fluorescence was designated F_{max}. Cells were then exposed to a similar solution, but now containing a Ca^{2+}-free solution that contained (in mM) 140 LiCl, 5 KCl, 1.2 KH_2PO_4, 1.2 $MgCl_2$, 0 $CaCl_2$, 20 HEPES, 4 EGTA, 0.005 Ionomycin, 0.01 CPA, 5 Caffeine and 1 Oubain and the resulting fluorescence was designated F_{min}. The following equation was used to convert Fluo-4 AM fluorescence readings F into $[Ca^{2+}]_i$:

$[Ca^{2+}]_i = Kd(F-F_{min})/(F_{max}-F)$

The Fluo-4 AM dissociation constant (K_d) of 345 nM was assumed according to the manufacturer's declaration. The area under the curve's analyses of the last 5 calibrated consecutive Ca^{2+}-transients were done using Peak Area (nM*ms) parameter from LabChart Pro V8's Peak Analysis package.

Western Blotting

For each western blots ventricle of 3 WT and 3 MDX age matched mice were used. Proteins were extracted from mouse hearts using Whole Cell Lysis Buffer (WCLB) (in mM: 20 Tris-HCl, 150 NaCl, 1 Na_2EDTA, 1 EGTA, 2.5 NaPyrophosphate, 1 NaVanadat, 1 PMSF, 1 DTT, 1% Triton, pH 7.5) and quantified using NanoDrop One using custom procedure at 290 nm wavelength. Protein separation was performed in 4-12% SDS-PAGE NuPAGE® Gel (Invitrogen) for dystrophin and 10% for nNOS, iNOs, eNOS and tubulin at 200V for 50 min using 1X NuPAGE® SDS Running Buffer for electrophoresis (Invitrogen; 50 mM MOPS, 50 mM Tris Base, 0.1% SDS, 1 mM EDTA, pH 7.7). Then the separated proteins were transferred into PVDF-membrane with 1X NuPAGE® Transfer Buffer (Invitrogen) with 20% methanol (in mM: 25 Bis-Tris, 25 Bicine, 1 EDTA, pH 7.2). Membranes were blocked with milk fat free 5% dissolved in 1X Tris-Buffer Solution (TBS; 100 mM TRIS-HCL, 1.5 M NaCl (1.50 M) and pH 7.4 with NaOH) with 1% Tween-20 (TBS-T). The following primary antibodies diluted in TBS-T were incubated overnight at 4°C: Dystrophin (Abcam, cat no. ab15277, 1:200); nNOS (Abcam, cat no. ab76067, 1:1000); iNOS (Abcam, cat no. ab15323, 1:40); eNOS (Abcam, cat no. ab50260, 1:1000); GAPDH (Abcam, cat no. ab 181603, 1:10,000). The secondary antibody was diluted in TBS-T and incubated for one hour at room temperature: Goat Anti-Rabbit IgG (Abcam, cat no. ab205718, 1:20,000). Secondary antibodies were coupled to HRP (Horse Redish Peroxidase) allowing signal detection with the AceGlow Chemiluminescence Substrate (VWR International GmbH, cat no. 730-1511).

Statistical Analysis

Only cells showing a normal behavior in Ca^{2+}-Transients as described here[130,131] at all 6 stimulations during measurements were used for the statistical analysis. Statistically significant outliers were detected by the free online available GraphPad QuickCalcs (α=0,05). Only those were removed from the data set and statistical analysis was performed with GraphPad Prism 7. For graphical presentation boxplots were chosen where the line in the box represented the median and the 25% and 75% quartiles by the extremes of the box. The whiskers reached from the minimum to the maximum. The data sets were statistically compared by ANOVA with Bonferroni post hoc pairwise multiple comparisons vs. control.
